# Exposure to Antibacterial Chemicals Is Associated With Altered Composition of Oral Microbiome

**DOI:** 10.3389/fmicb.2022.790496

**Published:** 2022-04-28

**Authors:** Hilde Kristin Vindenes, Huang Lin, Rajesh Shigdel, Tamar Ringel-Kulka, Francisco Gomez Real, Cecilie Svanes, Shyamal D. Peddada, Randi J. Bertelsen

**Affiliations:** ^1^Department of Occupational Medicine, Haukeland University Hospital, Bergen, Norway; ^2^Department of Clinical Science, University of Bergen, Bergen, Norway; ^3^Department of Biostatistics, Graduate School of Public Health, University of Pittsburgh, Pittsburgh, PA, United States; ^4^Department of Maternal and Child Care, University of North Carolina, Chapel Hill, NC, United States; ^5^Department of Gynaecology and Obstetrics, Haukeland University Hospital, Bergen, Norway; ^6^Centre for International Health, University of Bergen, Bergen, Norway; ^7^Biostatistics and Bioinformatics Branch, National Institute of Child Health and Human Development, Bethesda, MD, United States; ^8^Oral Health Center of Expertise in Western Norway, Bergen, Norway

**Keywords:** oral microbiome, chemicals, ANCOM-BC, RHINESSA, differential abundance

## Abstract

Antimicrobial chemicals are used as preservatives in cosmetics, pharmaceuticals, and food to prevent the growth of bacteria and fungi in the products. Unintentional exposure in humans to such chemicals is well documented, but whether they also interfere with human oral microbiome composition is largely unexplored. In this study, we explored whether the oral bacterial composition is affected by exposure to antibacterial and environmental chemicals. Gingival fluid, urine, and interview data were collected from 477 adults (18–47 years) from the RHINESSA study in Bergen, Norway. Urine biomarkers of triclosan, triclocarban, parabens, benzophenone-3, bisphenols, and 2,4- and 2,5-dichlorophenols (DCPs) were quantified (by mass spectrometry). Microbiome analysis was based on 16S amplicon sequencing. Diversity and differential abundance analyses were performed to identify how microbial communities may change when comparing groups of different chemical exposure. We identified that high urine levels (>75th percentile) of propyl parabens were associated with a lower abundance of bacteria genera *TM7 [G-3], Helicobacter, Megasphaera, Mitsuokella, Tannerella, Propionibacteriaceae [G-2]*, and *Dermabacter*, as compared with low propylparaben levels (<25^th^ percentile). High exposure to ethylparaben was associated with a higher abundance of *Paracoccus*. High urine levels of bisphenol A were associated with a lower abundance of *Streptococcus* and exposure to another environmental chemical, 2,4-DCP, was associated with a lower abundance of *Treponema, Fretibacterium*, and *Bacteroidales [G-2]*. High exposure to antibacterial and environmental chemicals was associated with an altered composition of gingiva bacteria; mostly commensal bacteria in the oral cavity. Our results highlight a need for a better understanding of how antimicrobial chemical exposure influences the human microbiome.

## Introduction

Antimicrobial chemicals are used as preservatives in cosmetics, pharmaceuticals, food, food-wrapping, and kitchen utensils (Dann and Hontela, 2011; Ferguson et al., 2017). The chemicals are added to the products to prevent the growth of bacteria and fungi (Calafat et al., 2010). Parabens have been used as preservatives in foods, drugs, and cosmetics for more than 50 years, and have antimicrobial properties, especially against fungi and Gram-positive bacteria. The mode of action for parabens is thought to be disruption of bacterial membrane transport processes (Freese et al., 1973) or inhibition of the DNA or RNA synthesis (Nes and Eklund, 1983). The antimicrobial effectiveness increases with the length of the alkyl chain. Butyl has the highest molecular weight and thus the longest alkyl chain, followed by propylparaben, ethylparaben, and methylparaben (Soni et al., 2005). Another antimicrobial compound, triclosan, prevents bacteria growth by inhibiting the bacteria's fatty acid synthesis (Russell, 2004).

The main routes of exposure to these chemicals are through the mouth or skin, with the most important sources being food and personal care products (Ye et al., 2007; Calafat et al., 2008; Philippat et al., 2015; Ferguson et al., 2017). Biomarkers of exposure to these chemicals can be measured in urine, which represents all routes of exposure (oral, inhaled, or dermal). In a previous analysis of this study population, urine levels of parabens were found to correlate strongly with the frequency of use of personal care products in both men and women (Vindenes et al., 2021). However, women report more frequent use of cosmetic products, corresponding to higher urinary levels of several phenols and paraben biomarkers than in men. Biomarkers of the environmental chemicals, bisphenols, and dichlorophenols were more commonly detected in urine from men (Vindenes et al., 2021). Bisphenol analogs are present in a variety of consumer products and foods (Liao and Kannan, 2014). The main source of exposure to bisphenols is believed to be leakage from the coating of metal cans coming into contact with food (Pacyga et al., 2019), whereas human exposure to the herbicides and pesticide chemicals (2,4- and 2,5-dichlorophenol) occur through air, water, and food (Ye et al., 2014).

Although it is well known that humans are extensively exposed to these broad-spectrum antibacterial chemicals, their interaction with the human microbiome is largely unexplored. Few studies have assessed the association between chemical exposure and oral microbiome. Stanaway and co-workers (Stanaway et al., 2017) reported that blood concentration of a non-antibacterial chemical, the insecticide Azinphos-methyl, leads to significant perturbation of common bacterial genera and less bacterial diversity in the buccal swab microbiome. In mice models, both methylparaben (Hu et al., 2016) and triclosan exposure were associated with an altered composition of gut microbiota, especially a lower abundance of Bifidobacteria, which has an anti-inflammatory effect (Hu et al., 2016; Gao et al., 2017; Yang et al., 2018). However, in a cross-over study with personal care products with and without triclosan and triclocarban, no significant effects of TCS or TCC exposure were observed for gut or oral microbiome composition (Poole et al., 2016). Although the intended use of the environmental chemicals, the bisphenols and dichlorophenols, is not to exert antibacterial effects, the 2,4-dichlorophenoxyacetic acid is used in the production of insecticides and herbicides, and therefore it is possible that dichlorophenols have some unintentional bactericidal effect (Jerschow et al., 2014).

The effect of exposure to different chemicals on the oral microbiome is not well known and data are scarce. This study aimed to assess whether the oral bacterial composition is affected by exposure to antibacterial or environmental chemicals in a general Norwegian population.

## Methods

### Study Population and Sample Collection

The study population includes 477 adult participants (≥18 years of age) investigated as part of the RHINESSA generation study (www.rhinessa.net) in Bergen, Norway. Data were gathered in 2014–2015 using questionnaires, interviews, and clinical examination. Weight and height were recorded during the clinical examination. We collected information regarding the use of antibiotics in the 4 weeks before the clinical examination, and characteristics like educational level and smoking. The questionnaires are available at www.rhinessa.net.

The clinical examination included urine and gingival fluid sampling. The time of day for collection of urine and gingival fluid samples was recorded, together with information on whether the participants were fasting before sample collection. Urine specific gravity was measured by a handheld Atago refractometer PAL 10-S (ATAGO Co., Ltd, Bellevue, WA, USA) before freezing the urine in polypropylene tubes (−80°C) without preservatives. Community periodontal index (CPI) was clinically assessed by a modified version of the WHO's guidelines (Ainamo et al., 1982), as previously described (Pérez Barrionuevo et al., 2018): CPI 0 for healthy gingiva; CPI 1 for gingival bleeding; CPI 2 for calculus; CPI 3 for periodontal pockets of 4–5 mm; and CPI 4 if periodontal pockets ≥6 mm. Ten index teeth were examined to screen periodontal status: from the upper jaw: 17, 16, 11, 26, and 27, and from the lower jaw: 47, 46, 31, 36, and 37. We also recorded if any of the 10 index teeth were missing. Gingival fluid was collected with sterile paper points (PROTAPER, Jacobsen Dental) from the gingival crevice (area between the gingiva crest and the neck of the tooth) at five per-protocol predetermined sites in the lower and upper jaws; between the two frontal teeth, left frontal tooth (lateral side), the right frontal tooth (lateral side), left molar number 6 (facing molar 5), and right molar number 6, facing molar number 5. Sterile procedures were applied with sterile mirrors and tweezers, gloves, and surgical face masks. The paper points were frozen directly (-80°C) after collection in 2 ml Microtubes safelock Biopur tubes without buffer; with five paper points (from the upper or the lower jaw) stored in one tube.

### Quantification of Urine Biomarkers of Chemical Exposure

Exposure to antibacterial and environmental chemicals were quantified as biomarkers in urine. The chemicals can broadly be categorized into three groups: (1) antibacterial/cosmetic chemicals: methyl-, ethyl-, butyl- and propylparabens, triclosan, triclocarban, and benzophenone-3 (the four parabens are also combined into one exposure variable based on the molar sum of the individual parabens); (2) bisphenols (plastic chemicals): bisphenol A, bisphenol S, and bisphenol F; and (3) herbicide/pesticide chemicals: 2,4- and 2,5-dichlorophenol. The urine samples were shipped on dry ice to CDC (Atlanta, GA, USA), where the total (free + conjugated) concentrations of the chemicals were quantified using a modification of the automated online solid-phase extraction high-performance liquid chromatography-tandem mass spectrometry method reported in Ye et al. (Ye et al., 2005). The limits of detection (LOD) varied between 0.11 and 2.35 and with machine-read values replacing the zeros for concentrations below LOD, as previously described (Vindenes et al., 2021).

### Bacterial DNA Extraction

Bacterial DNA was extracted from the gingival fluid samples and based on all five paper points from the lower jaw. The gingiva paper points were incubated at 37°C for 1 h with 200 μl of lysozyme solution in PBS and 0.2 g of glass beads. After adding 200 μl of ATL buffer with proteinase K, the samples were incubated overnight. The next day, the samples were vortexed on the tissue layer for 5 min, and then centrifuged for 3 min at full speed. A total of 350 μl was transferred to a new tube and the same amount of AL buffer was added and incubated for 10 min at 70°C. The same amount of 100% ethanol was added, and all were mixed, transferred on a spin column, and spun for 1 min at 13,200 x g. The samples were washed two times (AW1 buffer (500 μl) and 1-min spin and AW2 buffer (500 μl) and 2-min spin). Follow-throughs were discarded and the samples were air-dried for 10 min. Elution buffer was then added, and membranes were incubated for 3 min and spun for 1 min at 13,200 x g. Eluates amount of 20 μl and on-column purification was performed with minelute columns (Genesee).

### 16S rRNA Gene Amplicon Sequencing

Overall, 12.5 ng of total DNA was amplified using a combination (4:1) of Universal and *Bifidobacterium*-specific primers targeting the V1-V2 region of the bacterial 16S rRNA gene (Edwards et al., 1989; Fierer et al., 2008). Primer sequences contained overhang adapters appended to the 5' end of each primer for compatibility with the Illumina sequencing platform. The complete sequences of the primers were:

- 8F-5' TCGTCGGCAGCGTCAGATGTGTATAAGAGACAGAGAGTTTGATCCTGGCTCAG3'.
- BifidoF-5' TCGTCGGCAGCGTCAGATGTGTATAAGAGACAGAGGGTTCGATTCTGGCTCAG3', and
- 338R-5' GTCTCGTGGGCTCGGAGATGTGTATAAGAGACAGGCTGCCTCCCGTAGGAGT3'.

Master mixes contained 12.5 ng of total DNA, 0.2 μM of each primer, and 2x KAPA HiFi HotStart ReadyMix (KAPA Biosystems, Wilmington, MA, USA). The thermal profile for the amplification of each sample had an initial denaturing step at 95°C for 3 min, followed by the cycling of denaturing at 95°C for 30 s, annealing at 55°C for 30 s, and a 30-s extension at 72°C (25 cycles), a 5-min extension at 72°C and a final hold at 4°C. Each 16S amplicon was purified using the AMPure XP reagent (Beckman Coulter, Indianapolis, IN, USA). In the next step, each sample was amplified using a limited cycle PCR program, adding Illumina sequencing adapters and dual-index barcodes [index 1(i7) and index 2(i5)] (Illumina, San Diego, CA, USA) to the amplicon target. The thermal profile for the amplification of each sample had an initial denaturing step at 95°C for 3 min, followed by a denaturing cycle of 95°C for 30 s, annealing at 55°C for 30 s and a 30-s extension at 72°C (8 cycles), a 5-min extension at 72°C and a final hold at 4°C. The final libraries were again purified using the AMPure XP reagent (Beckman Coulter), quantified, and normalized before pooling. The DNA library pool was then denatured with NaOH, diluted with hybridization buffer, and heat-denatured before loading on the MiSeq reagent cartridge (Illumina) and the MiSeq instrument (Illumina). Automated cluster generation and paired-end sequencing with dual reads were performed according to the instructions of the manufacturer.

### Sequencing Data Analysis

Multiplexed paired-end fastq files were produced from the sequencing results of the Illumina MiSeq using the Illumina software configureBclToFastq. The paired-end fastqs were joined into a single multiplexed, single-end fastq using the software tool fastq-join. Demultiplexing and quality filtering were performed on the joined results. Quality analysis reports were produced using the FastQC software. Bioinformatics analysis of bacterial 16S amplicon sequencing data was conducted using the Quantitative Insight Into Microbial Ecology (QIIME) software (Caporaso et al., 2010). Operational taxonomic unit (OTU) picking was performed on the quality-filtered results using pick_de_novo_otus.py. Chimeric sequences were detected and removed using ChimeraSlayer. To assign taxonomy, we used the Human Oral Microbiome Database (www.homd.org), which is a library that contains genomes from the human oral cavity (Chen et al., 2010). A total of 47,443,921 reads were obtained for 477 subjects by V1-V2 16S RNA sequencing. An average number of 99,463 (from 10,017 to 292,207) reads per sample was used for downstream analysis. A total of 242 distinct bacterial operational taxonomic units (OTUs) were identified.

### Statistical Analysis

Descriptive statistics are presented as mean ± SD for continuous variables and as frequency (percentage) for categorical variables.

Different bacterial alpha diversity indices, namely, Shannon diversity index, Faith's phylogenetic diversity index (Faith's PD), and observed OTU count (at OTU level) were calculated to examine the associations with various population characteristics. Linear regression was implemented to test for statistical significance. In addition, the Shannon diversity index (a measure of richness, evenness, and divergence) was used to further investigate the association between alpha diversity at genus level and urine levels of chemical biomarkers, using Wilcoxon rank-sum test. For beta diversity (at genus level), Principal Coordinates Analysis (PCoA) was performed to visualize Bray–Curtis distance matrices. Permutational multivariate ANOVA (PERMANOVA) (Anderson and Walsh, 2013) and permutational analysis of multivariate dispersion (PERMDISP) (Anderson, 2006) were applied to compare the microbial community clustering between groups.

To test which taxa were differentially represented between the exposure groups, we performed a differential abundance analysis using analysis of compositions of microbiomes with bias correction (ANCOM-BC) (Lin and Peddada, 2020) at the genus level. This methodology is aimed at detecting differentially abundant microbes between two or more groups while controlling for other covariates.

We excluded participants (*n* = 14) who had used antibiotics in the last 4 weeks before gingival sampling from our main analyses, as the antibiotics are likely to affect the microbiome diversity. The Shannon diversity index was significantly lower (*p* = 0.004) for those reporting antibiotic use in the last 4 weeks before sampling compared to those who had not used antibiotics. In this study, the question of interest was to detect whether certain oral bacteria were differentially abundant with respect to varying exposure levels of chemicals. The chemicals were propylparaben (PPB), methylparaben (MPB), ethylparaben (EPB), butylparaben (BPB), the sum of parabens (molar sum of parabens), triclosan (TCS), triclocarban (TCC), benzophenone-3 (BP3), 2,4-dichlorophenols (2,4-DCP), 2,5-dichlorophenol (2,5-DCP), bisphenol A (BPA), bisphenol S (BPS), and bisphenol F (BPF). Each chemical was analyzed with a separate model while correcting for age, body mass index (BMI), smoking (never, ex-smoker, and current smoker), and time of day of sampling (urine and microbiome samples). Specifically, the chemicals for which biomarkers were detected in at least 50% of the urine samples, were categorized into four categories; Q1–Q4 represent the quartiles of the urine biomarker concentration of the chemicals. High chemical exposure refers to having a urine level above the 75th percentile (Q4). For chemicals for which the biomarkers were detected in <50% of the urine samples (BPB, TCS, TCC, and 2,5-DCP) we dichotomized the biomarkers according to whether they were below or above the limit of detection which is a commonly applied procedure (Savage et al., 2012; Quirós-Alcalá et al., 2018; Vindenes et al., 2021). For chemicals that were categorized into 4 groups, the ANCOM-BC global test was first implemented to identify OTUs that were differentially abundant in at least one group. *p* values obtained from the global test were denoted as “screening *p* values” and adjusted for multiple comparisons using the B-H procedure (Benjamini and Hochberg, 1995). Then correspondingly, to each chemical, for each OTU discovered significantly by the global test, the *p* values for each pairwise comparison among the four exposure groups (of each chemical) was derived from the ANCOM-BC pairwise test. Furthermore, we also performed a test for patterns, both increasing and decreasing, in the OTU abundance over the four exposure groups. *p* values from the pattern analysis were adjusted by the B-H procedure as well. For the dichotomized exposures, only the two-group comparisons were applied. STATA SE 17 (StataCorpLLC) and the R packages vegan, phyloseq, and ANCOM-BC were used for statistical analysis.

## Results

### Study Population Characteristics

**Figure 1:**
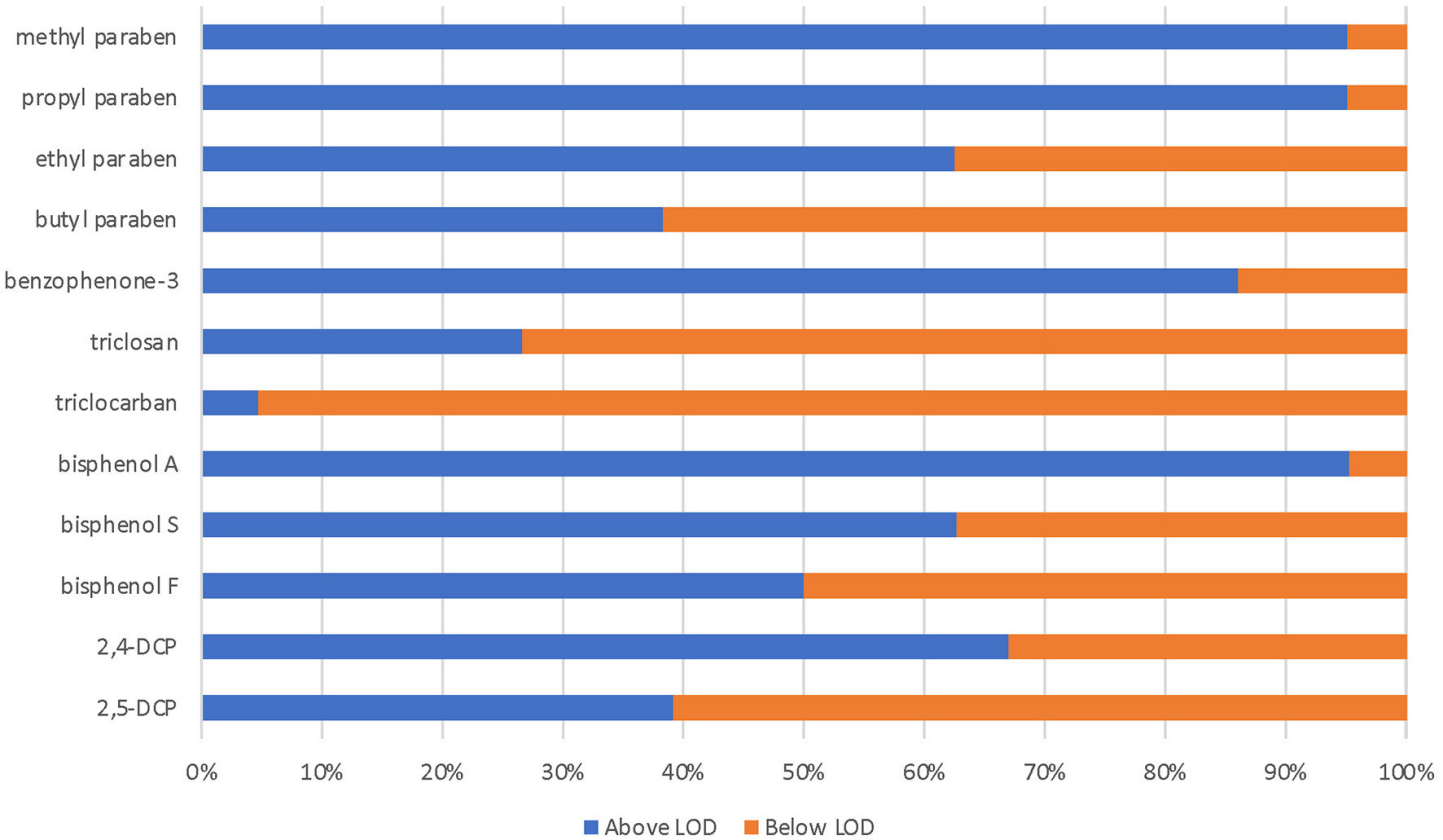
Percent of urine samples with urine biomarker above the limit of detection (LOD).

The women (*n* = 223) in the RHINESSA study in Bergen, Norway, were slightly younger than the men (*n* = 254) (mean age: 27.2 and 28.7 years, respectively). The women had a slightly higher educational level, lower BMI, and were less likely to be current smokers as compared to the male participants (Table 1). The chemicals show large variation in terms of the percentage of urine samples with detectable levels. The highest level of detection was found for MPB and PPB and BPA (95% detection), whereas triclocarban and triclosan were detectable (above LOD) in only 5% and 26% of the samples, respectively (Table 1, Figure 1). Urinary concentrations of parabens and TCS increased with the reported frequency of use of mouthwash (Table 2). CPI scores were available for 411 RHINESSA participants (10 teeth scored per person). Only 12 participants had a CPI score of 3 (4–5 mm periodontal pockets) and none had deeper pockets, and the majority of the study participants (82%) had a healthy periodontal state (CPI 0). Among the 411 participants, 27 (6.6%, 17 men and 10 women) had one or more missing tooth. In total, 44/4110 teeth (1.1%) were missing.

**Table 1:** Characteristics of study population (total, *n* = 477) and separately for women and men.

	**All** **(*n =* 477)**	**Women** **(*n =* 223)**	**Men** **(*n =* 254)**
**Age**			
Min–Max	18–47	18–45	18–47
Mean (SD)	27.98 ± 6.75	27.19 ± 6.57	28.67 ± 6.84
**BMI**			
Min–Max	17.08–46.50	17.20–42.89	17.08–46.49
Mean (SD)	25.07 ± 4.56	23.97 ± 4.50	25.97 ± 4.40
**Batch [n (%)]**			
Old	279 (58)	132 (59)	147 (58)
New	198 (42)	91 (41)	107 (42)
**Smoking [n (%)]**			
Never	288 (65)	144 (68)	144 (62)
Previous	106 (24)	50 (23)	56 (24)
Current	51 (11)	19 (9)	32 (14)
**Education [n (%)]**			
Primary	10 (2)	2 (1)	8 (3)
Secondary	179 (40)	74 (35)	105 (45)
Tertiary	259 (58)	138 (64)	121 (52)
**Ethylparaben (EPB) ng/mL**			
Minmax	0.08–2,957	0.13–2,957	0.078–706
Mean (SD)	20.56 (147)	33.85 (205)	8.73 (54.19)
**Propylparaben (PPB) ng/mL**			
Min–max	0.053–780	0.12–780	0.053–460
Mean (SD)	21.76 (66.08)	36.77 (82.59)	8.40 (42.67)
**Methylparaben (MPB) ng/mL**			
Min–max	0.21–3,726	0.78–3,726	0.21–1,355
Mean (SD)	102.96 (288)	166.12 (382)	46.73 (144)
**Butylparaben (BPB) ng/mL**			
Below LOD, n (%)	293 (63)	87 (40)	206 (84)
Above LOD, n (%)	172 (37)	132 (60)	40 (16)
**Molar sum of parabens ng/mL**			
Min–max	16.62–810,624	142–810,624	16.62–345,512
Mean (SD)	21,594 (57,495)	34,716 (75,438)	9,912 (30,098)
**Benzophenone-3 (BP3) ng/mL**			
Min–max	0.06–6,040	0.06–6,040	0.14–2,075
Mean (SD)	118 (517)	223 (726)	24.40 (137)
**Bisphenol A (BPA) ng/mL**			
Min–max	0.14–21.95	0.14–16.72	0.31–21.95
Mean (SD)	2.01 (2.29)	1.88 (2.22)	2.12 (2.35)
**Bisphenol F (BPF) ng/mL**			
Min–max	0.05–80.58	0.05–80.58	0.06–20.49
Mean (SD)	0.86 (4.16)	0.94 (5.63)	0.79 (2.14)
**Bisphenol S (BPS)**			
Min–max	0.037–8.36	0.042–3.14	0.037–8.36
Mean (SD)	0.23 (0.47)	0.23 (0.34)	0.23 (0.55)
**2,4-dichlorophenol (2,4-DCP) ng/mL**			
Min–max	0.04–7.45	0.04–3.14	0.04–7.45
Mean (SD)	0.24 (0.45)	0.23 (0.31)	0.25 (0.54)
**2,5-dichlorophenol (2,5-DCP) ng/mL**			
Below LOD, n (%)	285 (61)	154 (70)	131 (53)
Above LOD, n (%)	180 (39)	65 (30)	115 (47)
**Triclocarban (TCC) ng/mL**			
Below LOD, n (%)	444 (95)	208 (95)	236 (96)
Above LOD, n (%)	21 (5)	11 (5)	10 (4)
**Triclosan (TCS) ng/mL**			
Below LOD, n (%)	342 (74)	151 (69)	191 (78)
Above LOD, n (%)	123 (26)	68 (31)	55 (22)

*LOD, Limit of detection*.

*Information missing for smoking (n = 32), education (n = 29), chemical biomarkers (n = 12), and BMI (n = 1)*.

**Table 2:** Specific-gravity-adjusted urine chemical concentrations (ng/ml) by reported frequency of use of mouthwash for *n* = 464 participants with information available on reported frequency of use of mouthwash and available urine biomarker concentrations.

	**Reported frequency of use of mouthwash**				
**Urine biomarker**	**Never or <1/week** **Median [max]**	**Once week** **Median [max]**	**Once daily** **Median [max]**	**Twice daily** **Median [max]**	*p*-value^*^
Ethylparaben	1.45 [209]	1.71 [289]	2.22 [471]	3.35 [2,957]	0.1
Propylparaben	1.12 [780]	1.74 [460]	2.14 [532]	13.52 [331]	0.0002
Methylparaben	14.57 [1,824]	15.49 [1,148]	23.51 [3,269]	67.80 [3,726]	0.002
Butylparaben	0.10 [23]	0.14 [137]	0.10 [40]	0.17 [19]	0.1
Bisphenol A	1.33 [21.9]	1.33 [8.9]	1.31 [20.3]	1.41 [16.7]	0.8
Bisphenol F	0.26 [20.5]	0.27 [4.7]	0.30 [3.9]	0.36 [80.6]	0.5
Bisphenol S	0.15 [3.13]	0.15 [1.39]	0.15 [8.4]	0.16 [1.12]	0.8
Benzophenone-3	7.48 [4,525]	11.12 [2,156]	7.70 [6,040]	9.00 [2,075]	0.6
2,4 Dichlorophenol	0.16 [7.4]	0.16 [1.0]	0.15 [3.1]	0.19 [1.6]	0.1
2,5-Dichlorophenol	0.12 [15.0]	0.12 [1.69]	0.09 [4.1]	0.16 [0.4]	0.1
Triclocarban	0.06 [0.9]	0.07 [0.4]	0.06 [0.8]	0.10 [0.5]	0.5
Triclosan	0.95 [748]	1.04 [278]	1.11[860]	1.75 [49.4]	0.04

* *p value from Kruskal–Wallis for differences between urine concentrations by frequency of use of mouthwash*.

### Alpha and Beta Diversity, and Association With Urine Chemicals

Bacterial alpha diversities (Faith's phylogenetic diversity index and OTU count) were higher among men than women (Table 3). Faith's phylogenetic diversity index was lower for those who had not fasted on the day of sample collection and for gingival collection later in the day (Table 3), although no difference was observed by the other alpha-diversity indices such as Shannon diversity index (richness, evenness, and divergence) and OTU count. Shannon diversity and the OTU count decreased with increasing frequency of use of mouthwash (Table 3).

**Table 3:** Linear regression modeling for change (ß) in alpha-diversity indices (Shannon diversity index, Faith's PD, and OTU count) by population characteristics.

	**Shannon diversity index** **[ß±SD]**	^*^p–value	**Faith PD index** **[ß±SD]**	^*^p–value	**OTU count** **[ß±SD]**	^*^p–value
**Gender**						
Women	ref	0.3	ref	**<**0.001	ref	0.03
Men	0.05 ± 0.05		3.82 ± 1.04		16.13 ± 7.22	
**Fasting**						
Yes	ref		ref	0.05	ref	0.9
No	0.02 ± 0.06		−2.07 ± 1.06		−0.42 ± 7.36	
**Education**						
Primary school	ref	0.5	ref	0.97	ref	0.04
Secondary school	0.05 ± 0.19		−3.66 ± 3.62		−12.01 ± 25.75	
University or college	0.006 ± 0.19		−3.21 ± 3.59		−26.55 ± 25.54	
**Smoking**					
Never	ref	0.6	ref	0.9	ref	0.2
Previous	0.02 ± 0.07		−0.56 ± 1.23		2.84 ± 9.18	
Current	0.04 ± 0.09		0.10 ± 1.65		15.44 ± 12.31	
**Time of day of sample collection**					
Before 10 AM	ref	0.8	ref	0.007	ref	0.6
10 AM−12 PM	−0.05 ± 0.08		0.44 ± 1.54		−2.38 ± 11.15	
12–2 PM	0.06 ± 0.08		−1.89 ± 1.55		4.92 ± 11.21	
After 2 PM	−0.05 ± 0.09		−3.64 ± 1.77		−12.48 ± 12.80	
**BMI group**						
<18.5	−0.18 ± 0.21	0.2	−3.65 ± 4.10	0.1	−21.28 ± 28.33	4
≥18.5–25	ref		ref		ref	
≥25–30	0.12 ± 0.06		1.24 ± 1.20		8.45 ± 8.27	
≥30	0.40 ± 0.08		1.72 ± 1.64		2.86 ± 11.34	
**Mouthwash**						
Never	ref	0.03	ref	0.7	ref	0.02
Once a week	−0.02 ± 0.07		2.35 ± 1.35		−0.38 ± 9.28	
Once a day	−0.13 ± 0.07		−0.17 ± 1.32		−16.86 ± 9.09	
Twice a day	−0.17 ± 0.12		−1.96 ± 2.37		−32.40 ± 16.30	
**Use of fluoride toothpaste**					
Never	ref	0.8	ref	0.3	ref	0.9
Once a daily	0.06 ± 0.17		0.49 ± 3.32		18.44 ± 22.94	
Twice a day	−0. 04 ± 0.16		1.95 ± 3.03		7.54 ± 20.89	
>Twice a day	0.11 ± 0.19		2.63 ± 3.68		20.63 ± 25.36	

* *p value for linear association across all categories. Information missing for smoking (n = 32), education (n = 29), time of day of sample collection (n = 4), and BMI, use of mouthwash and fluoride toothpaste (n = 1)*.

**Figure 2:**
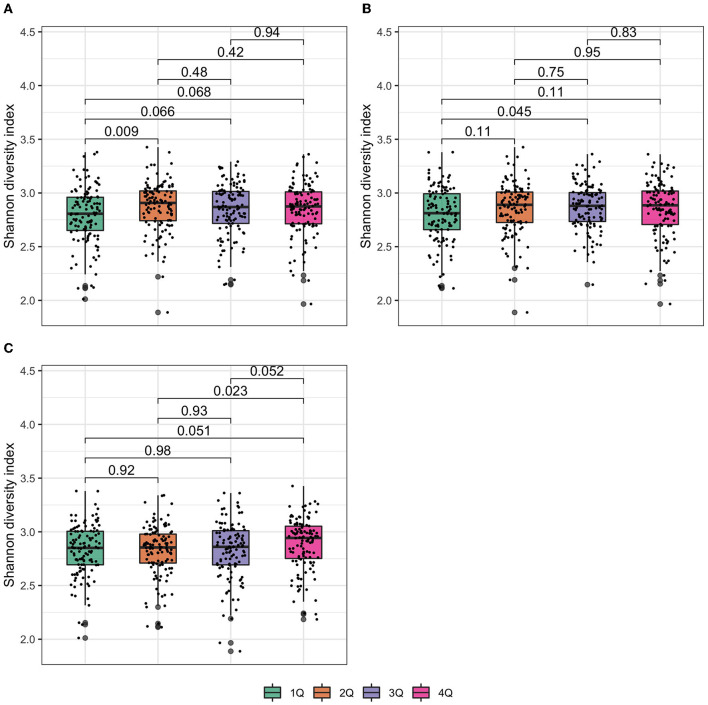
Box plot of Shannon diversity index at the genus level. In the box plot, the lower and upper hinges correspond to the first and third quartiles (the 25th and 75th percentiles). The median is represented by a solid line within the box. The upper whisker extends from the hinge to the largest value (maxima) no further than 1.5 times interquartile range (IQR, distance between the first and third quartiles) from the hinge, the lower whisker extends from the hinge to the smallest value (minima) at most 1.5 times IQR of the hinge. Data beyond the end of the whiskers are called “outlying” points. *N* = 477 samples examined over study groups (denoted by different colors) and the data points are overlaid in each box. *p* values were given by Wilcoxon rank-sum test. **(A)** Methylparaben; **(B)** propylparaben; and **(C)** benzophenone-3.

Shannon diversity index was not associated with the presence of urine biomarkers of exposure to TCS, BPB, TCC, and 2,5-DCP (Supplementary Figures 1A–D) but it increased with the increasing exposure of urine biomarker concentrations of MPB (Figure 2A). Similar patterns were observed for PPB (Figure 2B) and BP3 concentrations (Figure 2C).

**Figure 3:**
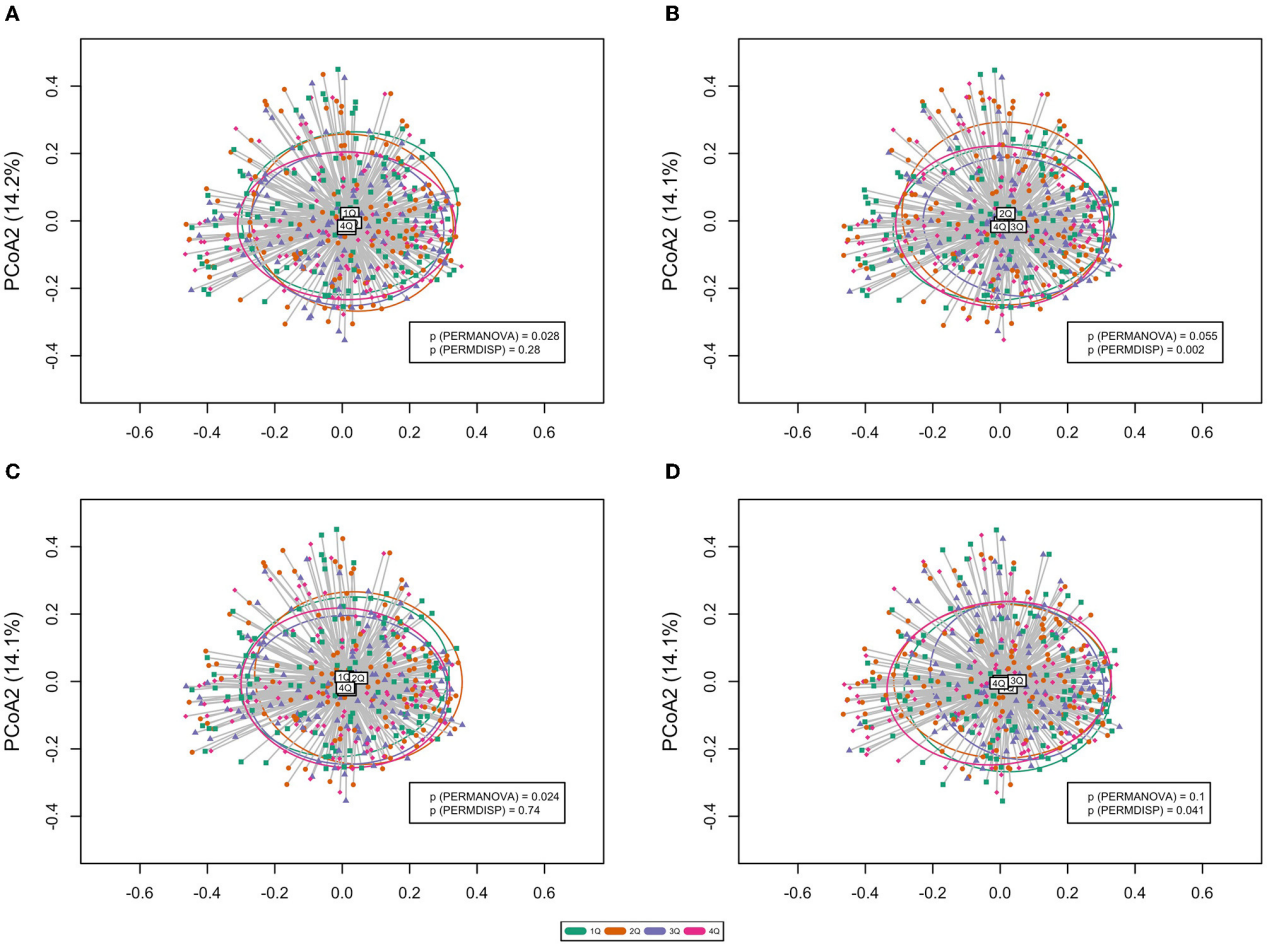
Principal coordinates analysis (PCoA) plot of the oral microbiome beta diversity (Bray–Curtis dissimilarity) at the genus level. Ellipses stand for 68% of data coverage. Lines are connected between samples and the corresponding group spatial median. *p* values from both PERMANOVA and PERMDISP tests are given in the plot. **(A)** Methylparaben; **(B)** propylparaben; **(C)** molar sum of parabens; and **(D)** 2,4-dichlorophenol.

Permutational multivariate ANOVA (PERMANOVA) test is often implemented to test for between-sample diversity (beta diversity) among microbes. Of note is that a significant PERMANOVA test either implies that the observed differences are due to different spatial medians or due to the heterogeneity of dispersions; therefore, a follow-up PERMDISP test is also performed to test the null hypothesis of “no difference in dispersion between groups.” Although beta diversities were not identified to be significantly different by most of the chemical exposures (Supplementary Figure 2), PERMANOVA and PERMDISP tests for microbial community clustering revealed a significant difference between different MPB exposures (Figure 3A), PPB exposures (Figure 3B), combined molar sum of parabens (Figure 3C), and 2,4-DCP (Figure 3D). It is clear to tell from the figures that the differences between exposures of MBP and combined parabens are driven by the location effect (different spatial medians), the difference between 2,4-DCP exposures is solely driven by heterogeneous dispersions (different group variations), and the difference between PPB exposures is determined by both the location and dispersion effects.

### Bacteria Taxa Distribution for Gingival Samples

**Figure 4:**
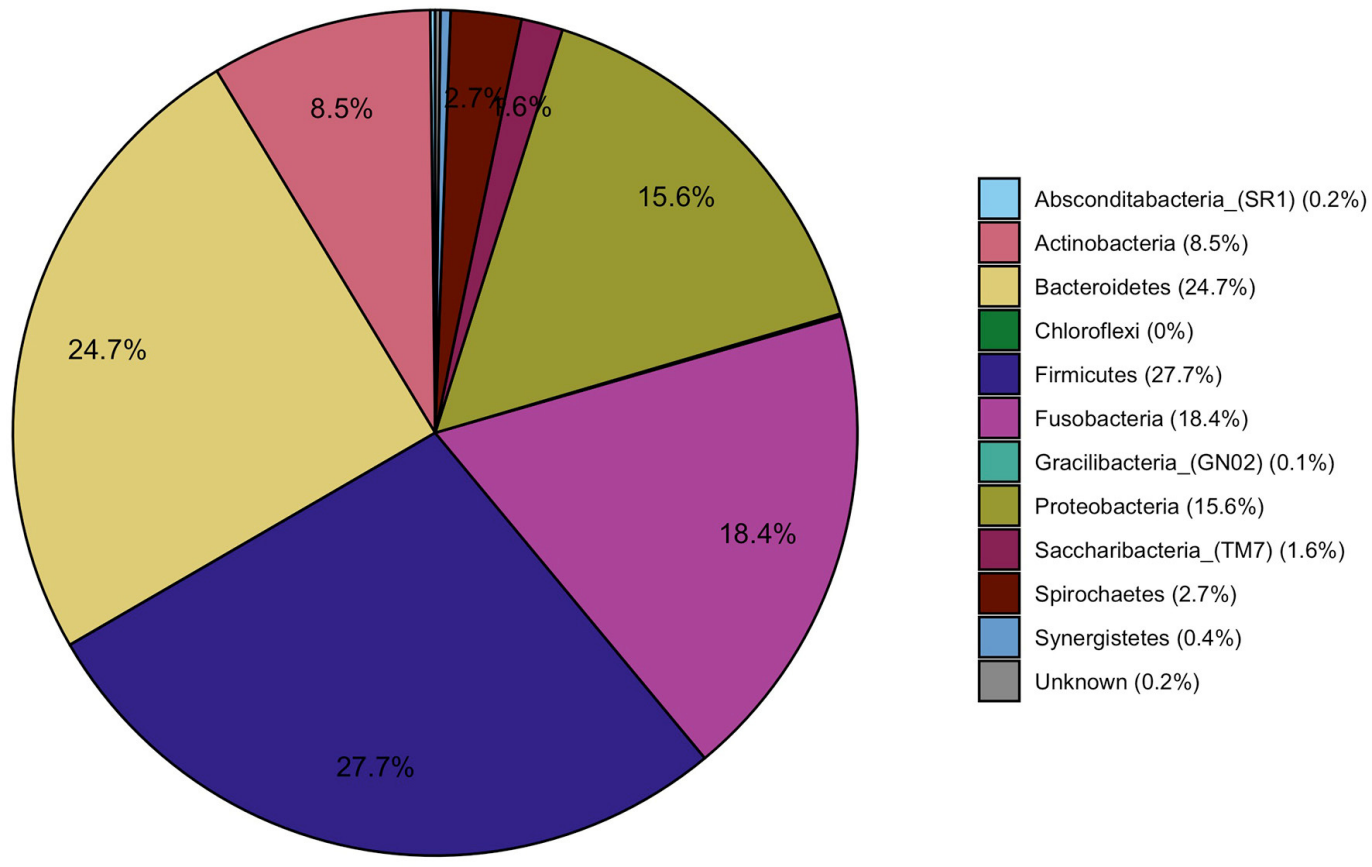
Pie charts representing the main phyla that constitute the oral microbiome in the study population.

The most prevalent bacterial phyla contributing to the gingival fluid samples were *Firmicutes* (27.7%), *Bacteriodetes* (24.7%), *Fusobacteria* (18.4%), *Proteobact*eria (15.6%), and *Actinobacteria* (8.5%) (Figure 4). At the genera level, *Fusobacterium (*15.2%, phylum*: Fusobacteria), Streptococcus (*9.7%, phylum: *Firmicutes), and Prevotella (*8.4%, phylum*: Bacteroidetes)* were the most prevalent bacteria and were present in all gingival samples (data not shown).

### ANCOM-BC Analyses for Antibacterial Chemicals and Gingival Fluid Microbiome

**Figure 5:**
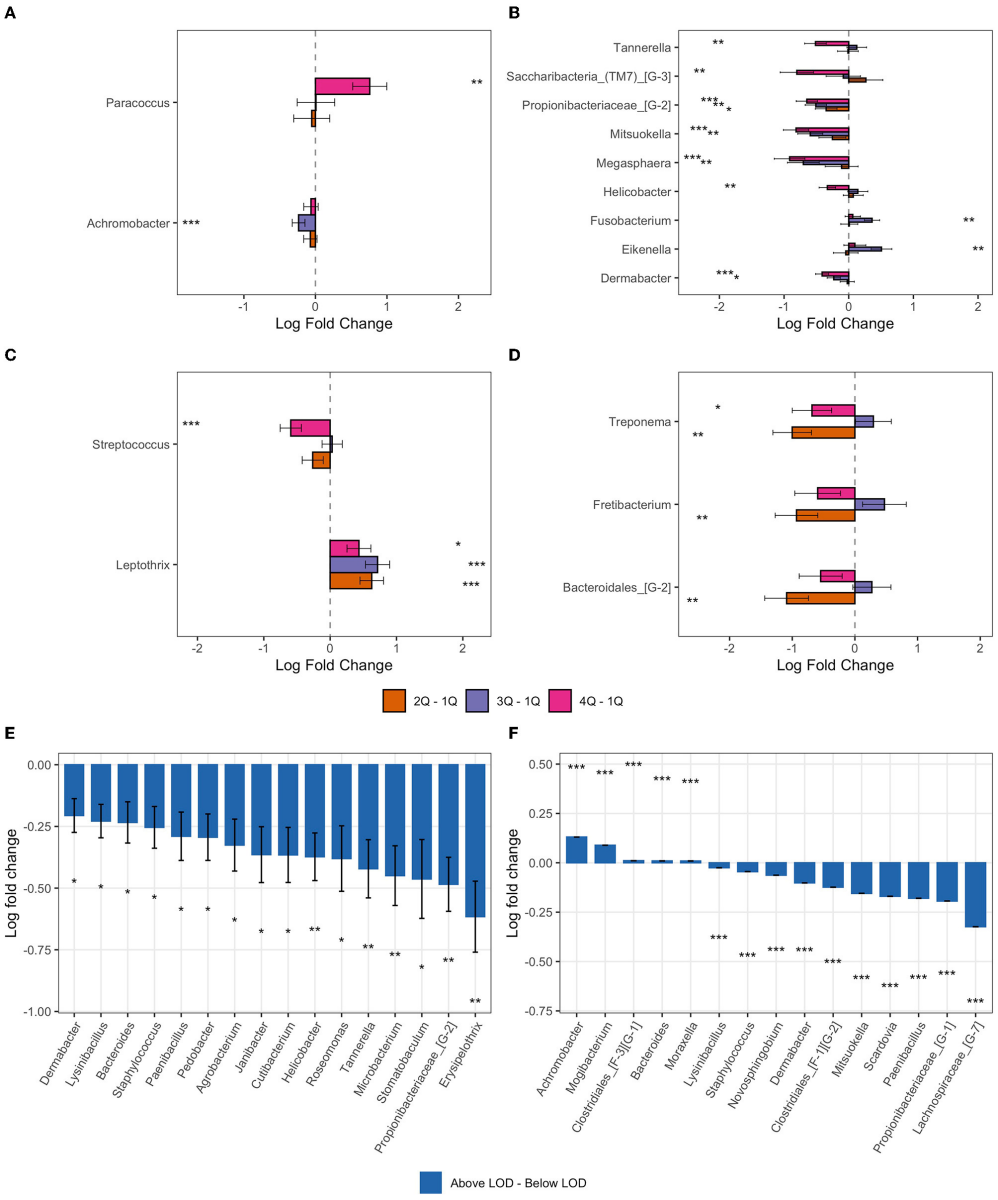
Bar plot of differentially abundant genera obtained from ANCOM-BC pairwise analysis. Data are represented by log-fold change (shown as a column) ± SE (shown as error bars). All log fold changes with *p* < 0.05 are indicated, *significant at 5% level of significance; **significant at 1% level of significance; and ***significant at 0.1% level of significance. **(A)** Ethylparaben; **(B)** propylparaben; **(C)** bisphenol A; **(D)** 2,4-DCP; **(E)** butylparaben; and **(F)** triclocarban.

ANCOM-BC methodology was applied to determine taxa that were differentially abundant among the four chemical exposure categories (quartiles of urine biomarker concentrations) adjusted for age, BMI, smoking, and time of day of collection of the urine and gingival samples. The pairwise testing of the 2nd, 3rd, and 4th quartile exposure group compared to the reference group (1st quartile and the lowest urine concentration) showed a significantly higher abundance (*p* < 0.01) in genus *Paracoccus* for the highest exposure to EPB exposure group compared to the lowest exposure group (Table 4, Figure 5A). High exposure to PPB was associated with a lower abundance of the genera *Dermabacter, Megasphaera, Mitsuokella, TM7 [G-3], Tannerella, Propionibacteriaceae [G-2]*, and *Helicobacter* as compared to the lowest level of PPB exposure (Table 4, Figure 5B). Another ubiquitous chemical present in almost all urine samples is BPA, for which high exposure was associated with a lower abundance of *Streptococcus* spp (Table 4, Figure 5C). In our study population, the genus *Streptococcus* contributed to as much as 9.7% of the gingival microbiome and was present in all the gingival samples. The exposure of 2,4-DCP was associated with a statistically significant lower abundance of *Treponema* in the 2nd and 4th quartiles and with *Fretibacterium* and *Bacteriodales [G-2]* in the 2nd quartile (Table 4, Figure 5D). *Treponema* belongs to the phylum *Spirochaetes*, and the 2.7% mean contribution in the relative abundance of *Treponema* to the overall genera composition (data not shown) corresponds to the 2.7% contribution of the phylum *Spirochaetes* to the overall phyla composition across all samples (Figure 4). Likewise, the genus *Fretibacterium* (from the phylum *Synergistetes*) contributed 0.4% to the overall genera composition which corresponds to the 0.4% contribution of *Synergistetes* to the overall phyla composition. Thus, chemical exposure is associated with altered taxon composition also on the phylum level.

**Table 4:** Pairwise test comparing the corresponding group with the reference group (1st quartile) of urine chemical biomarker exposure and bacterial genera.

**Chemical**	**Significant taxa [genus (P: phylum)]**	**Screening p–value**	**2nd Q−1st Q**	**3rd Q−1st Q**	**4th Q−1st Q**	**Samples with genera present (%)**
			**LFC (SE)**	**LFC (SE)**	**LFC (SE)**	
Ethyl paraben	*Achromobacter (P: Proteobacteria)*	0	−0.071 (0.094)	**−0.236 (0.089)** *^*b*^*	−0.063 (0.101)	10.5
	*Paracoccus (P: Proteobacteria)*	0.038	−0.052 (0.252)	0.006 (0.262)	**0.758 (0.238)** *^*b*^*	97.9
Propyl paraben	*Dermabacter (P: Actinobacteria)*	0.005	−0.023 (0.109)	**−0.232 (0.102)** *^*a*^*	**−0.411 (0.101)** *^*b*^*	10.9
	*Megasphaera (P: Firmicutes)*	0.005	−0.111 (0.255)	**−0.703 (0.246)** *^*b*^*	**−0.916 (0.237)** *^*b*^*	46.3
	*Mitsuokella spp (P: Firmicutes)*	0.005	−0.252 (0.209)	**−0.596 (0.194)** *^*b*^*	**−0.814 (0.196)** *^*b*^*	16.3
	*Saccharibacteria (TM7) [G−3] [P: Saccharibacteria (TM7)]*	0.007	0.266 (0.262)	−0.085 (0.266)	**−0.803 (0.258)** *^*b*^*	67.7
	*Eikenella (P: Proteobacteria)*	0.011	−0.046 (0.191)	**0.505 (0.161)** *^*b*^*	0.095 (0.170)	98.5
	*Tannerella (P: Bacteroidetes)*	0.023	−0.013 (0.160)	0.124 (0.152)	**−0.516 (0.167)** *^*b*^*	99.7
	*Fusobacterium (P: Fusobacteria)*	0.029	0.009 (0.132)	**0.360 (0.117)** *^*b*^*	0.062 (0.119)	100
	*Propionibacteriaceae [G−2] (P: Actinobacteria)*	0.029	**−0.349 (0.168)** *^*a*^*	**−0.509 (0.165)** *^*b*^*	**−0.649 (0.165)** *^*b*^*	24.7
	*Helicobacter (P: Proteobacteria)*	0.033	0.071 (0.154)	0.141 (0.155)	**−0.330 (0.128)** *^*a*^*	12.1
Bisphenol A	*Leptothrix (P: Fusobacteria)*	0.021	**0.628 (0.177)** *^*b*^*	**0.715 (0.182)** *^*b*^*	**0.435 (0.180)** *^*a*^*	41.1
	*Streptococcus (P: Firmicutes)*	0.021	−0.264 (0.158)	0.031 (0.153)	**−0.596 (0.160)** *^*b*^*	100
2,4–DCP	*Treponema (P:Spirochaetes)*	0.009	**−1.000 (0.308)** *^*b*^*	0.296 (0.285)	**−0.686 (0.315)** *^*a*^*	94.1
	*Fretibacterium (P: Synergistetes)*	0.031	**−0.911 (0.342)** *^*b*^*	0.462 (0.350)	−0.587 (0.367)	68.1
	*Bacteriodales [G−2] (P: Bacteroidetes)*	0.040	**−1.089 (0.349)** *^*b*^*	0.273 (0.304)	−0.546 (0.343)	88.7

*^a^p < 0.05; ^b^p < 0.01. Adjusted for age, smoking, BMI, education, and time of day of sampling collections. ANCOM-BC global test (whether each genus is differentially abundant in at least one quartile of urine chemical biomarker exposure) was used to obtain the screening p values. For each pairwise comparison, log fold change (LFC, natural log) and SE is given together with p values if statistically significant different from 1st quartile. Statistically significant values are highlighted in bold*.

*p values are not provided for nonsignificant associations (p > 0.05)*.

**Figure 6:**
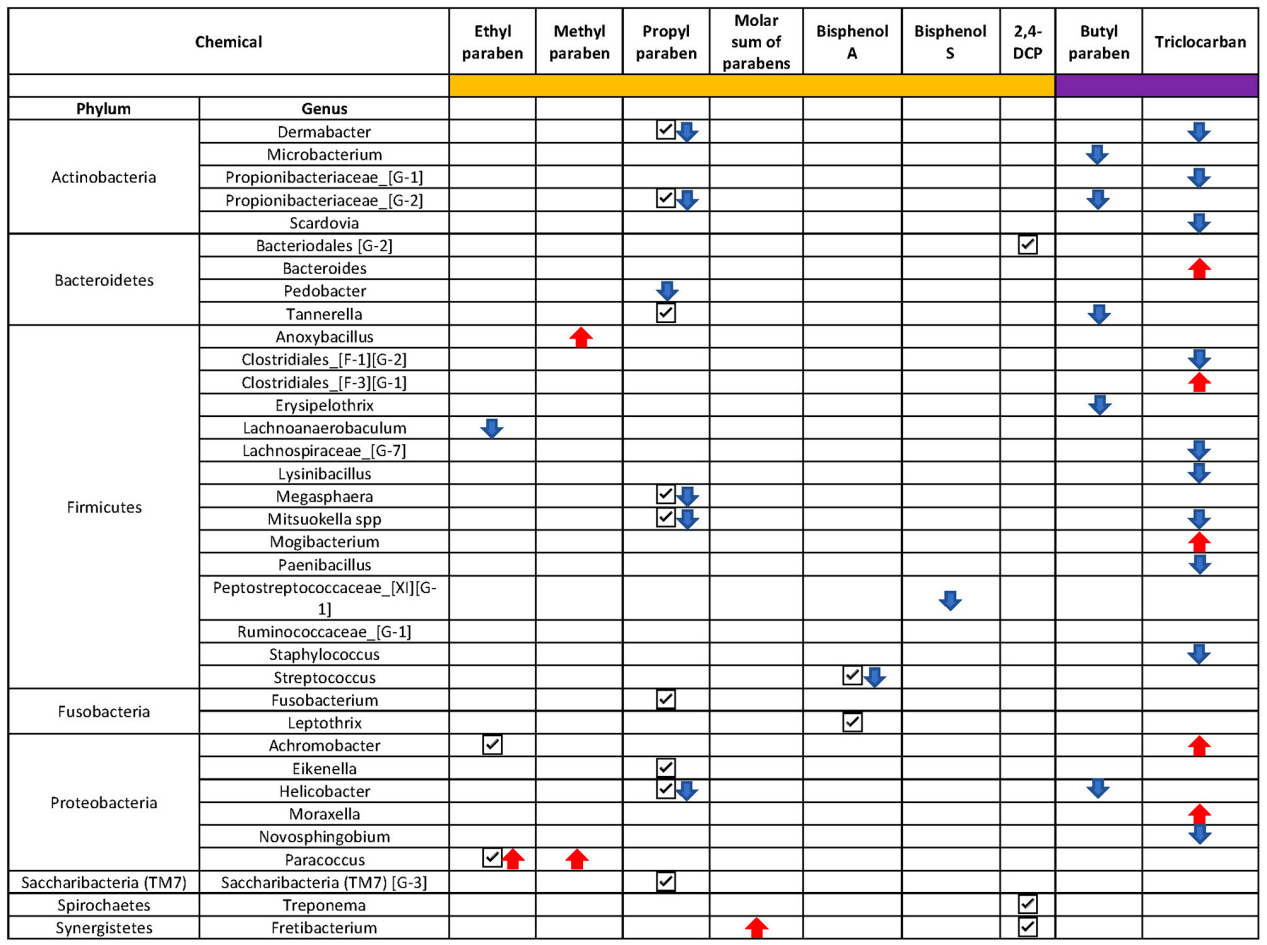
Overview of the ANCOM-BC results. Only taxa with a *p* < 0.01 were presented. Chemicals with sidebar color in orange were categorized into quartiles and chemicals with sidebar color in purple were categorized into below and above the limit of detection. Check mark “**✓**”: taxon was identified to be differentially abundant in at least one quartile of the chemical; downward arrow “
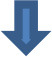
”: taxon was identified to be monotonically decreasing as the increase of the chemical concentration; upward arrow “
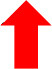
”: taxon was identified to be monotonically increasing as the chemical concentration increased.

In addition to the pairwise testing of the quartiles, we also tested whether there was a monotonic trend across the quartiles. In line with our findings from the pairwise comparisons, a monotonic decrease in *Streptococcus spp*. abundance was observed with an increase in exposure to BPA (*p* < 0.001) (Supplementary Figure 3A) and *Peptostreptococcaceae* [XI] [G-1] decreased monotonically (*p* < 0.001) with an increase in BPS exposure (Figure 6, Supplementary Figure 3B). The genera *Dermabacter, Propionibacteriaceae [G-2], Mitsuokella, Megasphera, Lachnoanaerobaculum*, and *Pedobacter spp* decreased monotonically with an increase in PPB concentrations (Figure 6, Supplementary Figure 3C). Increasing exposure to MPB was associated with increased abundance of *Anoxybacillus* and *Paracoccus spp*; with a doubling from 1st to 4th quartile and a 1-fold (natural log) decrease in TM7 from 1st to 4th quartile (Supplementary Figure 3D). An increase in *Fretibacterium spp* and decrease in *TM7 [G-3]* and *Ruminococcaceae [G-1] spp* was observed for increasing exposure to all parabens combined (Figure 6, Supplementary Figure 3E), whereas genus *Lachnoanaerobaculum* decreased and *Paracoccus* spp increased with increasing exposure to EPB (Supplementary Figure 3F).

Compared to urine samples with no detectable level of butyl parabens, biomarkers of exposure to BPB were associated with a lower abundance of several bacteria genera; five of these taxa had a *p* < 0.01 (Figures 5E, 6). These included some of the taxa identified to be associated with PPB exposure as well, namely, *Helicobacter, Tannerella*, and *Propionibacteriaceae [G-2]*. The same was observed for triclocarban (Figure 5F and Figure 6). However, only 5% of the participants had detectable levels of triclocarban, thus the size of the exposure groups is fairly small, and the results should be interpreted with caution.

## Discussion

This study found that urine biomarkers of antimicrobial and environmental chemicals are associated with an altered composition of the oral microbiome. High exposure to PPB, which is known to have antimicrobial effects, and exposure to BPB which is the paraben that most effectively kills bacteria were associated with a lower abundance of several commensal oral bacteria genera, such as *Dermabacter, Tannerella, Propionibacteriaceae [G-2]*, and *Helicobacter spp*. Increasing exposure to the ubiquitous environmental chemical, BPA, was associated with a lower abundance of *Streptococcus*.

To the best of our knowledge, this is the first study to report associations between urine biomarkers of antimicrobial and environmental chemicals and oral microbiome composition. The oral microbiome is characterized by a long-term stable microbiome (Rasiah et al., 2005) and is the body site with the greatest amount of core taxon (defined as taxa shared among 95% or more of the individuals) as compared to other body sites such as gut or skin (Moon and Lee, 2016). The healthy oral microbiome is affected by host-specific factors such as age and ethnicity and most likely genetics (Mason et al., 2013).

We found that microbial diversity changes little by population characteristics, except for use of mouthwash and gender. We noted that alpha diversities, namely, Faith's phylogenetic diversity index and OTU count are slightly higher in men as compared to women. There are two potential reasons that could lead to this heterogeneity. The most important aspect is the unbalanced distribution of periodontal diseases between men and women. Although we lack information on periodontal disease, it is known that crude periodontal status markers, such as CPI, can serve as a surrogate endpoint for periodontal health. Based on data from this study population, we have previously reported a higher CPI score among males and for current smokers (Pérez Barrionuevo et al., 2018). Furthermore, it is well-known that smoking affects periodontal health (Barbour et al., 1997), and evidence for the association between smoking and chronic periodontitis has been demonstrated in diverse populations (Nociti et al., 2015).

In this study, we did not find statistically significant differences in smoking status between men and women. Therefore, it is possible that the higher alpha diversities observed for men are due to the slightly higher CPI scores as compared to women. Results showed a higher bacterial diversity in participants reporting frequent gum-bleeding when brushing teeth and in those with the highest CPI score (Bertelsen et al., in revision, Journal of Clinical Periodontology).

There is a trend of increasing alpha diversity in the overweight (BMI 25-30) compared to groups with normal BMI (18.5-24), in line with previous studies on the oral microbiome and obesity (Yang et al., 2019). Together with the knowledge that phenolic chemicals can be stored in adipose tissue (Artacho-Cordon et al., 2017), we adjusted for BMI in our models when assessing associations between urine chemical biomarker levels and microbiome composition. Furthermore, exposure to antibacterial and environmental chemicals had little influence on the overall diversity in the oral samples. On the other hand, we found that the typical antibacterial chemicals that are used as preservatives in a range of cosmetics and personal care products, the parabens, were associated with the differential abundance of several bacteria genera.

The main contributing source for human exposure to parabens and other antimicrobial chemicals are personal care products (Bledzka et al., 2014), but both MPB and PPB are permitted for use as direct food additives and as indirect food additives in food-packaging materials (Soni et al., 2001, 2002). Urine biomarker levels of exposure to parabens correlate well with the reported frequency of use of personal care products (Vindenes et al., 2021). Furthermore, we found that the urine biomarker levels of parabens increased with the reported frequency of use of mouthwash, similar to reports from the NHANES study (Ferguson et al., 2017). In this study, we found that several bacterial taxa were less abundant among those with high concentrations of a urinary biomarker of propyl paraben and for those exposed to butyl parabens; many of these bacterial phyla belong to the *Firmicutes* and *Proteobacteri*a. Whereas, with increasing exposure to EPB, there was an increase in the genus *Paracoccus*, also a member of the Proteobacteria phylum, but a monotonic decrease in the abundance of *Lachnoanaerobaculum* spp (from the *Firmicutes* phylum). *Paracoccus spp* is present in almost all the gingival samples (98%) and thus is likely to be part of the commensal microbiome of the oral cavity. BPB is the most efficient of the parabens and therefore only minor amounts of this compound can be added to consumer products, mirrored by the low percentage of urine samples with detectable levels in this study. Nevertheless, detectable urine levels of this chemical were associated with decreasing relative abundance of a total of 16 different bacteria genera.

A lower abundance of *Bacteroidales [G-2]* and *Treponema* was associated with a higher level of 2,4-DCP, both are anaerobic Gram-negative bacteria, and have species that are associated with periodontitis (Kumar et al., 2003). *Treponema* is also a known member of the “red complex” associated with periodontal disease (Socransky et al., 1998). Another bacteria associated with the environmental chemical, 2,4-DCP, was *Fretibacterium*, a Gram-negative (obligatory anaerobic) bacillus dependent on other oral bacteria to grow. Putative periodontopathogens, such as *Fretibacterium* and *Treponema*, are often present at low levels in healthy subjects, but if the subgingival nutritional environment changes, taxa implicated in periodontitis may increase in abundance with concomitant decreases in health-associated species (Naginyte et al., 2019). The intended use of 2,4-DCP is not as a preservative, but rather primarily in the production of phenoxy acid herbicides such as 2,4-diphenoxyacetic acid and in the synthesis of pharmaceuticals and antiseptics. However, DCPs are found to lower bacterial numbers in marine plankton communities (Kuiper and Hanstveit, 1984), and 2,4-DCP is a known degradation product of triclosan in the environment.

Triclosan (TCS) is one of the best-known antimicrobial agents. However, due to the effectiveness of the chemical and its potential to induce antibiotic-resistant bacteria (Carey and Mcnamara, 2014), it is being phased out from many products. This may be the reason for the low number of samples with measurable amounts of TCS in this study (27%) as compared to our previous publication from a Norwegian population with 47% of the samples having detectable levels (Bertelsen et al., 2013). In this study, we did not find differences in gingival bacterial taxon between participants with and without detectable levels of urine biomarkers of TCS.

Increasing exposure to BPA was associated with decreasing abundance of *Streptococcus* spp. *Streptococcus* is one of the principal bacteria genera found in the healthy oral cavity and contributes to almost 10% of the overall abundance. Many of the numerous species of *Streptococcus* are commensals and some are associated with caries development (Corby et al., 2005).

The strengths of this study are the well-defined study population with state-of-the-art measurements of both urine chemicals and microbiome in gingival samples, and the relatively large number of study participants considering the literature within the field of microbiome. The gingival samples were analyzed at two different time points, but by the same laboratory technician and with the same methodology. Although we cannot rule out a potential batch effect, we have overcome this limitation by applying a novel biostatistical model, ANCOM-BC (Lin and Peddada, 2020) which takes into account the compositional nature of the data (Gloor et al., 2017) and which also controls for potential batch effect. This is one of the first studies where the ANCOM-BC biostatistical approach has been applied. ANCOM-BC allows for reporting directionality and effect size for abundances and at the same time control for covariates in the models. The model also takes into consideration multiple testing. The ANCOM-BC pattern analysis is still under development and so less reliable than those of pairwise tests but gives an innovative aspect to our article.

A limitation of this study is the simplified dental protocol and sampling procedure. This was necessary to enable a uniform collection of samples that can easily be adopted in any of our study centers and by field workers without dental training. Unfortunately, we do not have information on recent scaling or root planning, which could potentially have an impact on the gingival bacteria community. We collected information on the use of any antibiotics but did not ask about dental prophylaxis, in particular. Participants who had used antibiotics during the last 4 weeks were excluded from the analyses. We are unable to distinguish between periodontally healthy, gingivitis, and periodontitis cases and the CPI score may only provide indications on periodontal pocketing and inflammation. However, only 12 participants had a CPI score of 3 and none had a score of 4, and the majority (82%) were considered periodontally healthy when using CPI as a surrogate marker for periodontal status.

The use of primers should not impact the interpretation of the results. However, the choice of the V1-V2 primer set may have implications for comparison with other studies which have used the standard V3-V4 primer set. Still, the V1-V2 primer set may in many cases be a better fit than the V3-V4 primers (Kameoka et al., 2021). In particular for oral microbiota, the use of V1-V2 (V3) region primers is more suitable than the V3-V4 region, providing greater phyla type richness and evenness than V3-V4 and therefore gives a more representative assessment of the population diversity and community for oral bacterial genera (Zheng et al., 2015).

Urine was collected as a spot sample; a single spot of urine sample has previously been found to be representative of a person's daily exposure to parabens (Fisher et al., 2017). Although some of the compounds have a relatively short half-life, the daily use of personal care products and often continued use of the same cosmetic brands lead to continuous exposure to the compounds (Bertelsen et al., 2014; Guidry et al., 2015).

## Conclusions

Parabens have been used for decades and are still widely used as a preservative in foods and personal care products. This is despite regulations limiting the quantity of parabens allowed to be used in products for humans. Our findings suggest that oral microbiome composition was altered in relation to antibacterial and environmental chemical exposure. Our results highlight a need for a better understanding of how cosmetics and environmental chemical exposure influence the human microbiome and the potential clinical relevance of our findings.

## Data Availability Statement

The datasets presented in this study can be found in online repositories. The Dryad repository, accessible at: https://doi.org/10.5061/dryad.r2280gbfh; GitHub, accessible at: https://github.com/FrederickHuangLin/Oral-Microbiome-and-Chemical-Exposure.

## Ethics Statement

Ethical approval was obtained from the Regional Committee for Medical and Health Research Ethics in Western Norway (approval number #2012/1077). The patients/participants provided their written informed consent to participate in this study.

## Author Contributions

HV, HL, RS, and RB analyzed the data. RB, HV, HL, and SP drafted the article. HV, HL, SP, CS, and RB participated in the study design. HV, CS, FR, TR-K, and RB participated in the coordination and collection of data and providing the data. All authors were involved in critically discussing the results, revising the manuscript, and approval of the final manuscript.

## Funding

This project was funded by the Research Council of Norway (Grants Nos. 230827 and 273838) and the Western Norwegian Regional Health Authorities (Grant No. 912128). The Bergen RHINESSA study was funded by the Research Council of Norway (Grants Nos. 214123 and 228174) and the Western Norwegian Regional Health Authorities (Grants Nos. 912011, 911892, and 911631). This project has received funding from the European Research Council (ERC) under the European Union's Horizon 2020 research and innovation program (Grant Agreement No. 804199). The research of SDP was funded in part by the Intramural Research Program 429 of the *Eunice Kennedy Shriver* National Institute of Child Health and Human Development (NICHD), 430 National Institutes of Health, Bethesda, USA.

## Conflict of Interest

The authors declare that the research was conducted in the absence of any commercial or financial relationships that could be construed as a potential conflict of interest.

## Publisher's Note

All claims expressed in this article are solely those of the authors and do not necessarily represent those of their affiliated organizations, or those of the publisher, the editors and the reviewers. Any product that may be evaluated in this article, or claim that may be made by its manufacturer, is not guaranteed or endorsed by the publisher.

## Abbreviations

ANCOM-BC: Analysis of compositions of microbiomes with bias correction
LOD: Limit of detection
BMI: Body mass index
PCoA: Principal coordinates analysis
PERMANO: Permutational multivariate analysis of variance
PERMDISP: Permutational analysis of multivariate dispersion
PPB: Propylparaben
MPB: Methylparaben
EPB: Ethylparaben
BPB: Butylparaben
molar, TCS: Triclosan
TCC: Triclocarban
BP3: Benzophenone-3
2, 4-DCP, 2: 4-Dichlorophenols
2, 5-DCP, 2: 5-Dichlorophenol
BPA: Bisphenol A
BPS: Bisphenol S
and BPF: Bisphenol F.
