# Supplementary material for: Exposure to Antibacterial Chemicals Is Associated With Altered Composition of Oral Microbiome
Source: Front Microbiol. 2022 Apr 28;13:790496. doi: 10.3389/fmicb.2022.790496 (PMC9096491; doi:10.3389/fmicb.2022.790496)

Supplementary Figure 1. Box plot of Shannon diversity index at genus level. In the box plot, the lower and upper hinges correspond to the first and third quartiles (the 25th and 75th percentiles). The median is represented by a solid line within the box. The upper whisker extends from the hinge to the largest value (maxima) no further than 1.5 times Interquartile Range (IQR, distance between the first and third quartiles) from the hinge, the lower whisker extends from the hinge to the smallest value (minima) at most 1.5 times IQR of the hinge. Data beyond the end of the whiskers are called “outlying” points. N = 477 samples examined over study groups (denoted by different colors) and the data points are overlaid in each box. P-values were given by Wilcoxon rank-sum test. **a)** triclosan; **b)** butylparaben; **c)** triclocarban; **d)** 2,5-DCP; **e)** ethylparaben; **f)** molar sum of parabens; **g)** 2,4-dichlorophenol; **h)** bisphenol A; **i)** bisphenol S; **j)** bisphenol F.

a

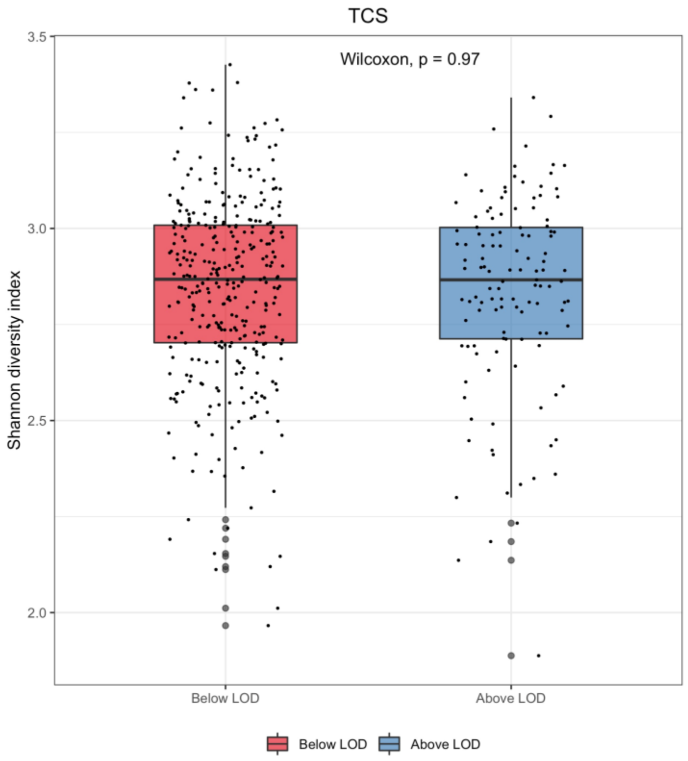

b

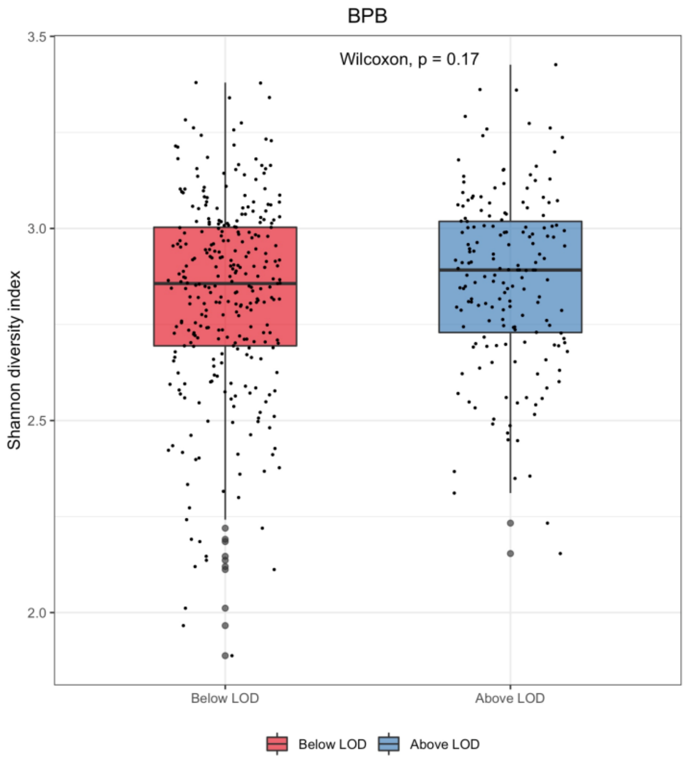

Supplementary Figure 1. Box plot of Shannon diversity index at genus level. In the box plot, the lower and upper hinges correspond to the first and third quartiles (the 25th and 75th percentiles). The median is represented by a solid line within the box. The upper whisker extends from the hinge to the largest value (maxima) no further than 1.5 times Interquartile Range (IQR, distance between the first and third quartiles) from the hinge, the lower whisker extends from the hinge to the smallest value (minima) at most 1.5 times IQR of the hinge. Data beyond the end of the whiskers are called "outlying" points. N = 477 samples examined over study groups (denoted by different colors) and the data points are overlaid in each box. P-values were given by Wilcoxon rank-sum test. **a)** triclosan; **b)** butylparaben; **c)** triclocarban; **d)** 2,5-DCP; **e)** ethylparaben; **f)** molar sum of parabens; **g)** 2,4-dichlorophenol; **h)** bisphenol A; **i)** bisphenol S; **j)** bisphenol F.

c

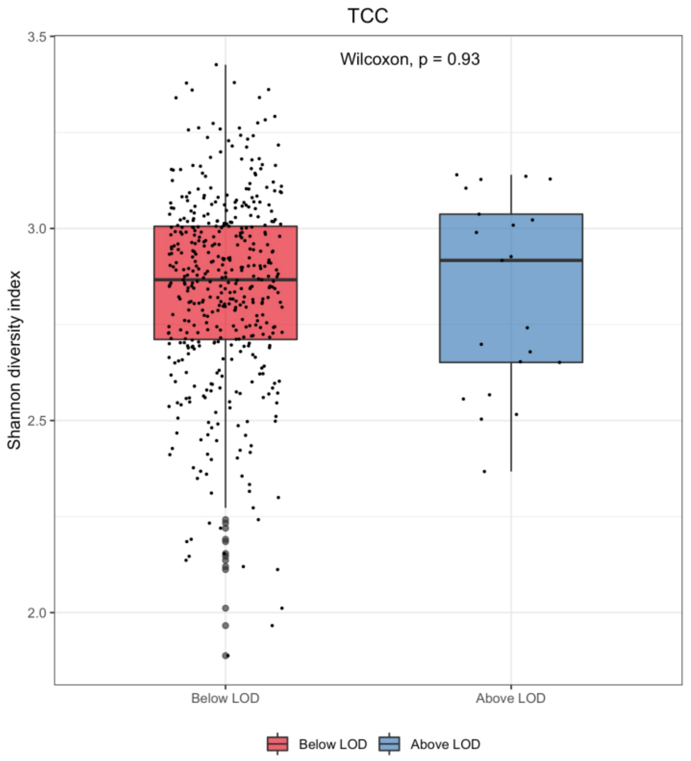

d

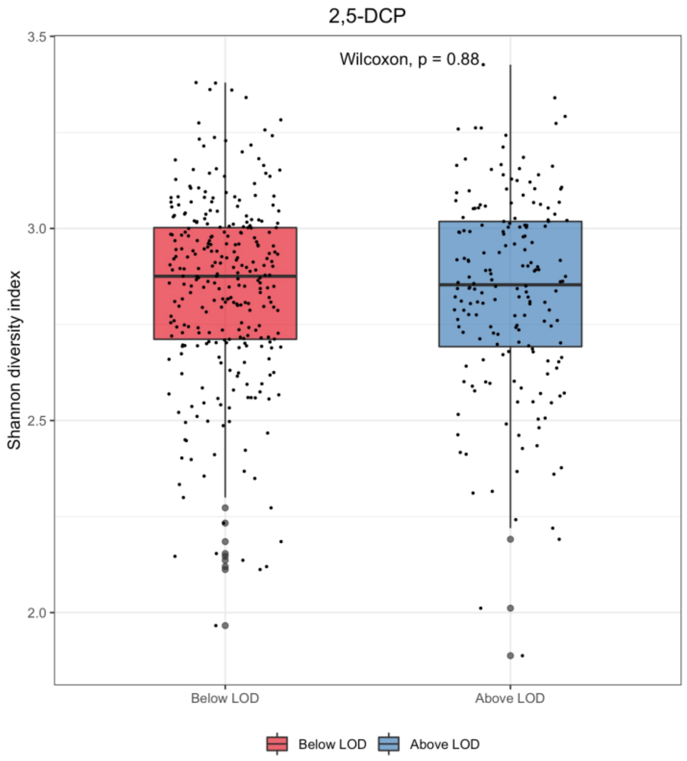

Supplementary Figure 1. Box plot of Shannon diversity index at genus level. In the box plot, the lower and upper hinges correspond to the first and third quartiles (the 25th and 75th percentiles). The median is represented by a solid line within the box. The upper whisker extends from the hinge to the largest value (maxima) no further than 1.5 times Interquartile Range (IQR, distance between the first and third quartiles) from the hinge, the lower whisker extends from the hinge to the smallest value (minima) at most 1.5 times IQR of the hinge. Data beyond the end of the whiskers are called "outlying" points. N = 477 samples examined over study groups (denoted by different colors) and the data points are overlaid in each box. P-values were given by Wilcoxon rank-sum test. **a)** triclosan; **b)** butylparaben; **c)** triclocarban; **d)** 2,5-DCP; **e)** ethylparaben; **f)** molar sum of parabens; **g)** 2,4-dichlorophenol; **h)** bisphenol A; **i)** bisphenol S; **j)** bisphenol F.

e

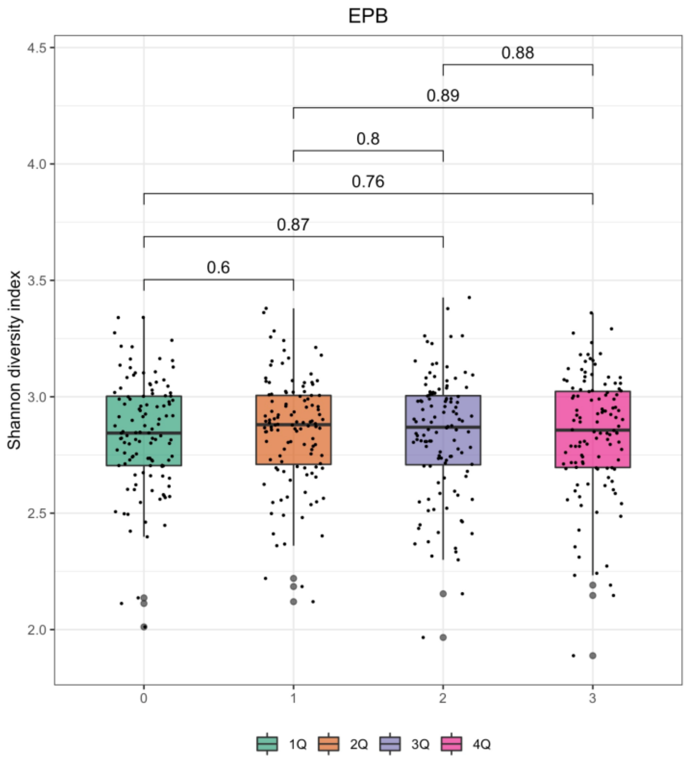

f

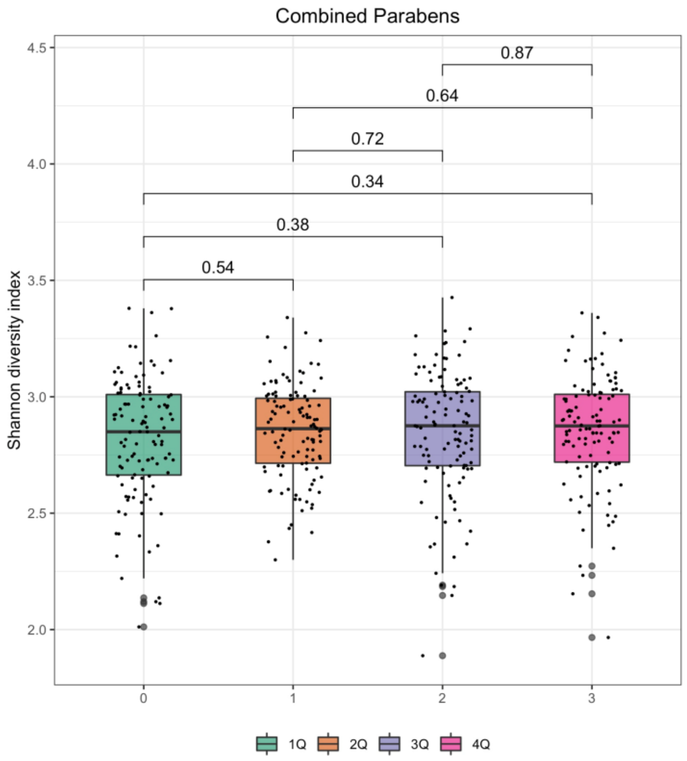

Supplementary Figure 1. Box plot of Shannon diversity index at genus level. In the box plot, the lower and upper hinges correspond to the first and third quartiles (the 25th and 75th percentiles). The median is represented by a solid line within the box. The upper whisker extends from the hinge to the largest value (maxima) no further than 1.5 times Interquartile Range (IQR, distance between the first and third quartiles) from the hinge, the lower whisker extends from the hinge to the smallest value (minima) at most 1.5 times IQR of the hinge. Data beyond the end of the whiskers are called "outlying" points. N = 477 samples examined over study groups (denoted by different colors) and the data points are overlaid in each box. P-values were given by Wilcoxon rank-sum test. **a)** triclosan; **b)** butylparaben; **c)** triclocarban; **d)** 2,5-DCP; **e)** ethylparaben; **f)** molar sum of parabens; **g)** 2,4-dichlorophenol; **h)** bisphenol A; **i)** bisphenol S; **j)** bisphenol F.

g

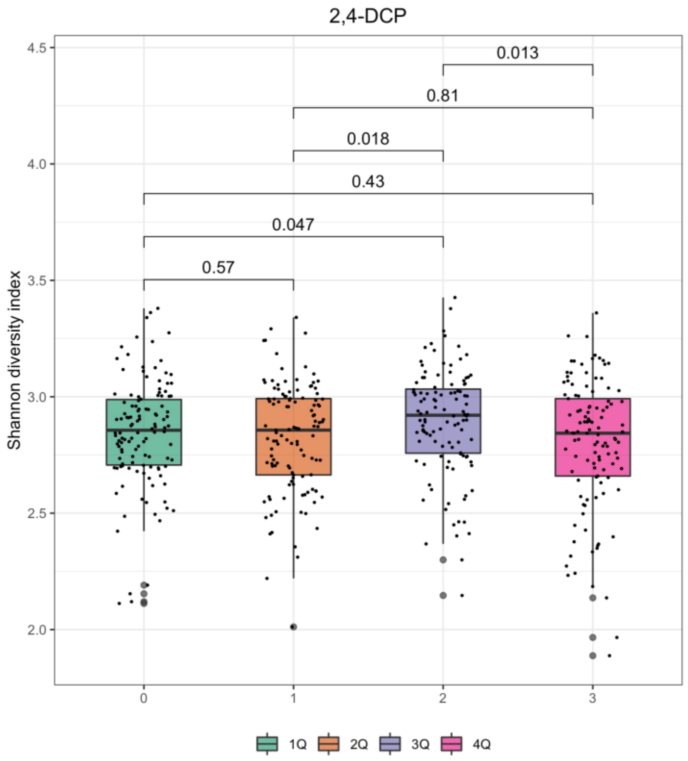

h

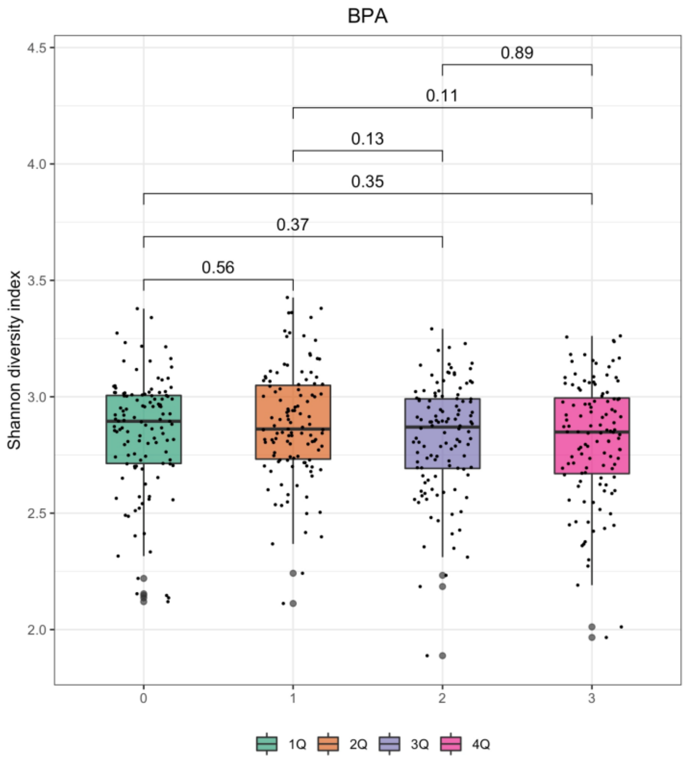

Supplementary Figure 1. Box plot of Shannon diversity index at genus level. In the box plot, the lower and upper hinges correspond to the first and third quartiles (the 25th and 75th percentiles). The median is represented by a solid line within the box. The upper whisker extends from the hinge to the largest value (maxima) no further than 1.5 times Interquartile Range (IQR, distance between the first and third quartiles) from the hinge, the lower whisker extends from the hinge to the smallest value (minima) at most 1.5 times IQR of the hinge. Data beyond the end of the whiskers are called “outlying” points. N = 477 samples examined over study groups (denoted by different colors) and the data points are overlaid in each box. P-values were given by Wilcoxon rank-sum test. **a)** triclosan; **b)** butylparaben; **c)** triclocarban; **d)** 2,5-DCP; **e)** ethylparaben; **f)** molar sum of parabens; **g)** 2,4-dichlorophenol; **h)** bisphenol A; **i)** bisphenol S; **j)** bisphenol F.

i

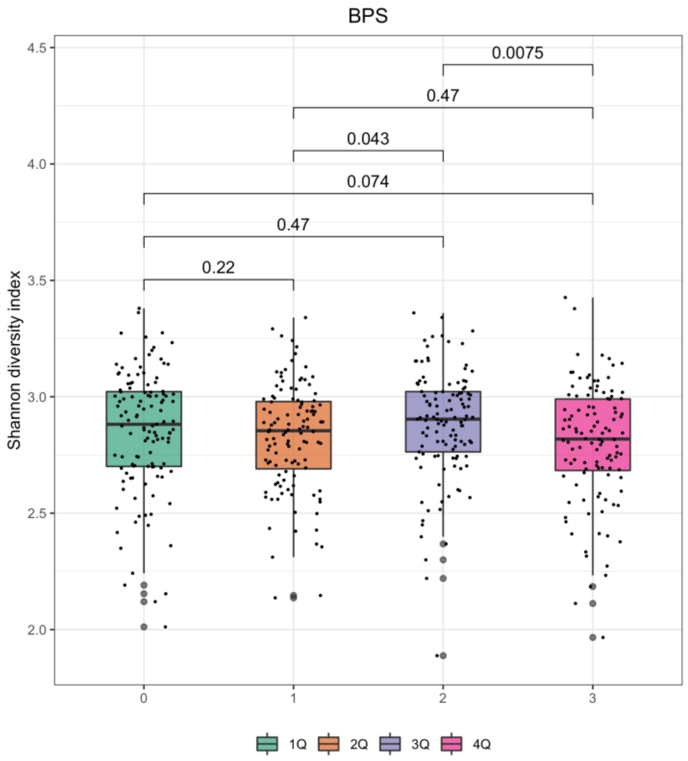

j

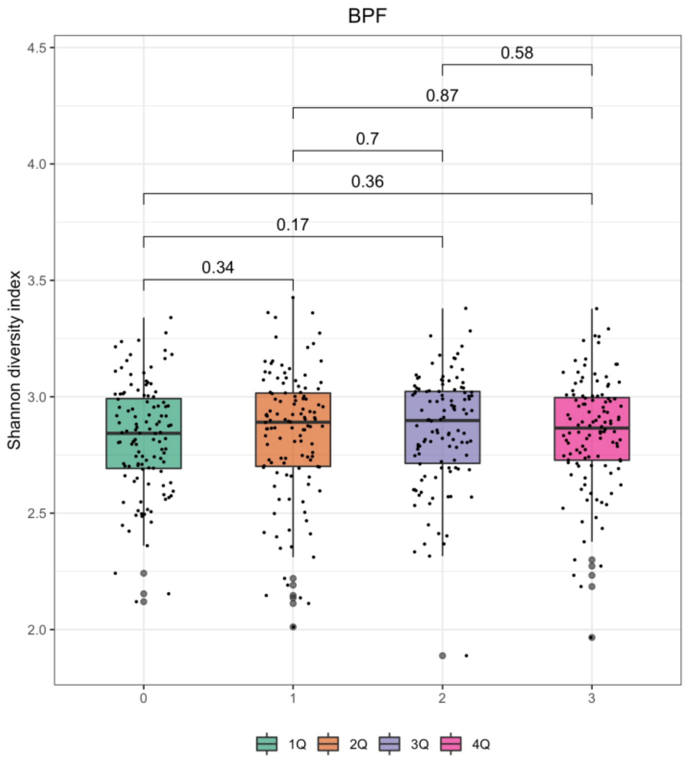

Supplementary Figure 2. Principal Coordinates Analysis (PCoA) plot of the fecal microbiome beta diversity (Bray-Curtis dissimilarity) at genus level. Ellipses stand for 68% of data coverage. Lines are connected between samples and the corresponding group spatial median. P-values from both PERMANOVA and PERMDISP tests are given in the plot. **a)** triclosan; **b)** butylparaben; **c)** triclocarban; **d)** 2,5-DCP; **e)** ethylparaben; **f)** benzophenone-3 ; **g)** bisphenol A; **h)** bisphenol F; **i)** bisphenol S.

a

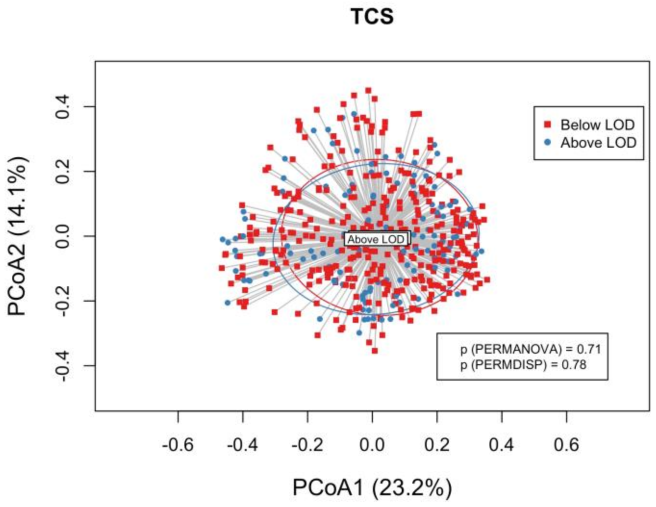

b

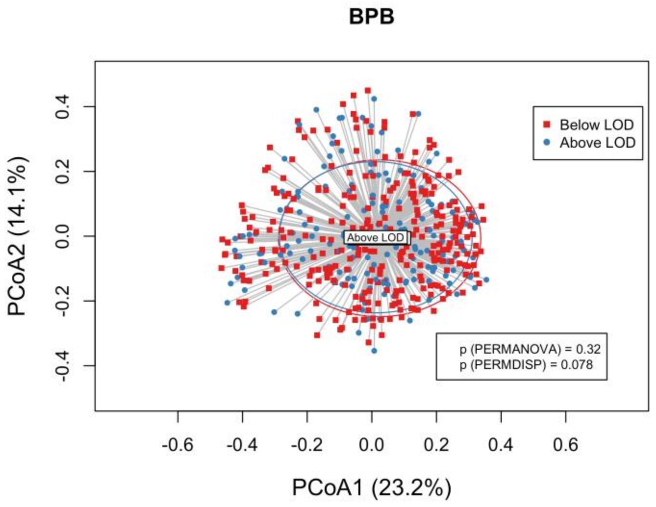

Supplementary Figure 2. Principal Coordinates Analysis (PCoA) plot of the fecal microbiome beta diversity (Bray-Curtis dissimilarity) at genus level. Ellipses stand for 68% of data coverage. Lines are connected between samples and the corresponding group spatial median. P-values from both PERMANOVA and PERMDISP tests are given in the plot. **a)** triclosan; **b)** butylparaben; **c)** triclocarban; **d)** 2,5-DCP; **e)** ethylparaben; **f)** benzophenone-3 ; **g)** bisphenol A; **h)** bisphenol F; **i)** bisphenol S.

c

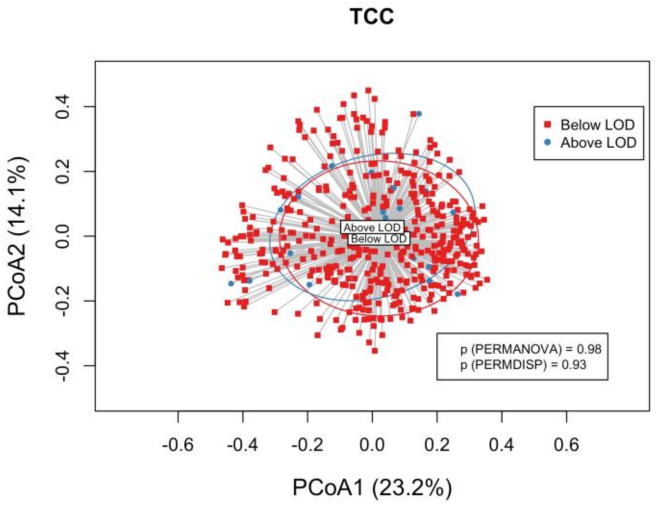

d

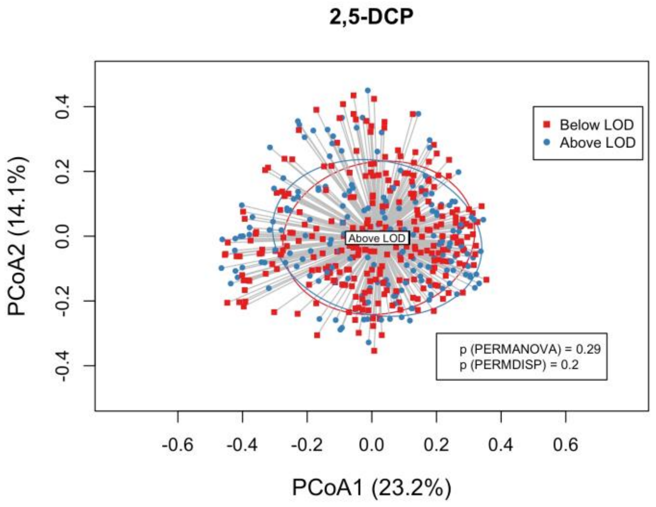

Supplementary Figure 2. Principal Coordinates Analysis (PCoA) plot of the fecal microbiome beta diversity (Bray-Curtis dissimilarity) at genus level. Ellipses stand for 68% of data coverage. Lines are connected between samples and the corresponding group spatial median. P-values from both PERMANOVA and PERMDISP tests are given in the plot. **a)** triclosan; **b)** butylparaben; **c)** triclocarban; **d)** 2,5-DCP; **e)** ethylparaben; **f)** benzophenone-3 ; **g)** bisphenol A; **h)** bisphenol F; **i)** bisphenol S.

e

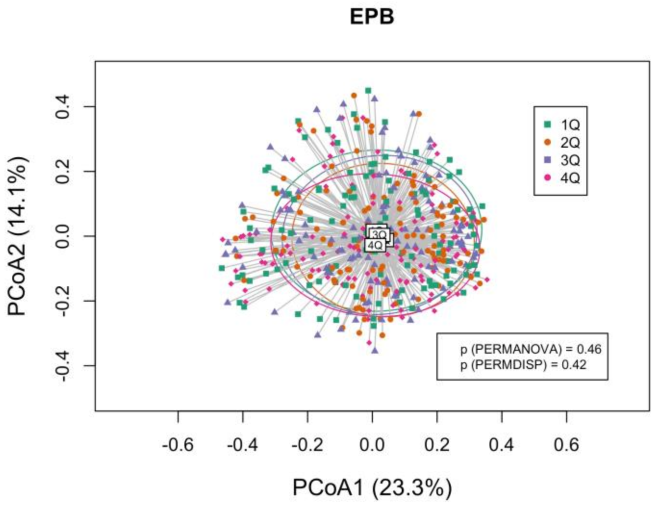

f

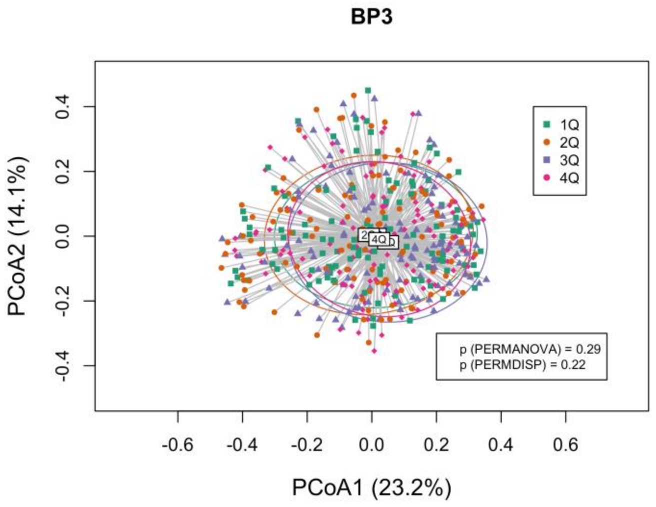

Supplementary Figure 2. Principal Coordinates Analysis (PCoA) plot of the fecal microbiome beta diversity (Bray-Curtis dissimilarity) at genus level. Ellipses stand for 68% of data coverage. Lines are connected between samples and the corresponding group spatial median. P-values from both PERMANOVA and PERMDISP tests are given in the plot. **a)** triclosan; **b)** butylparaben; **c)** triclocarban; **d)** 2,5-DCP; **e)** ethylparaben; **f)** benzophenone-3 ; **g)** bisphenol A; **h)** bisphenol F; **i)** bisphenol S.

g

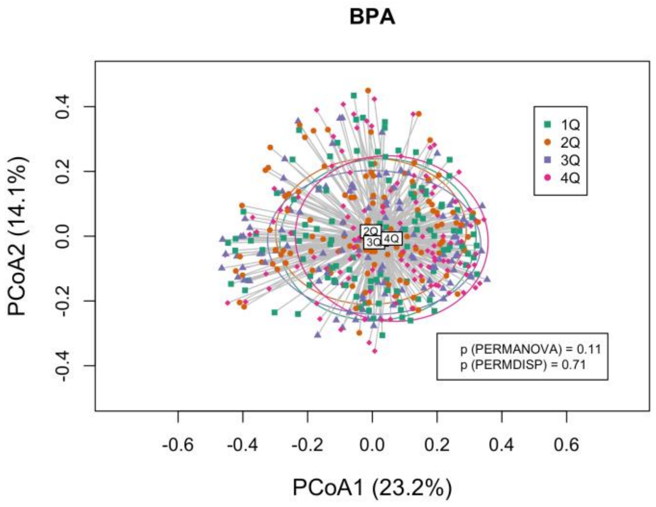

h

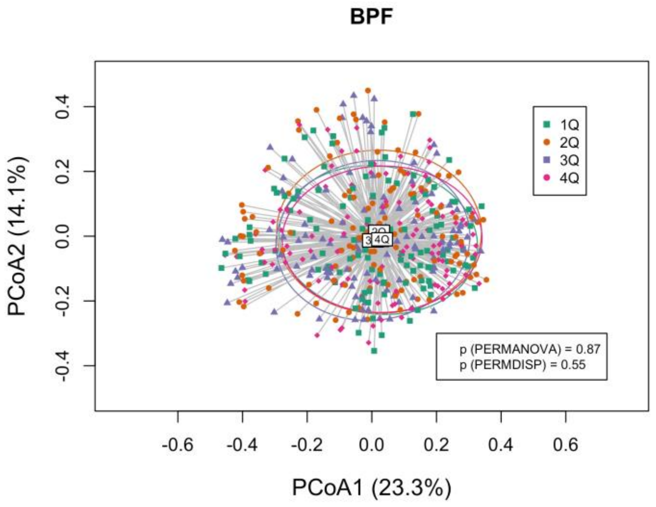

Supplementary Figure 2. Principal Coordinates Analysis (PCoA) plot of the fecal microbiome beta diversity (Bray-Curtis dissimilarity) at genus level. Ellipses stand for 68% of data coverage. Lines are connected between samples and the corresponding group spatial median. P-values from both PERMANOVA and PERMDISP tests are given in the plot. **a)** triclosan; **b)** butylparaben; **c)** triclocarban; **d)** 2,5-DCP; **e)** ethylparaben; **f)** benzophenone-3 ; **g)** bisphenol A; **h)** bisphenol F; **i)** bisphenol S.

i

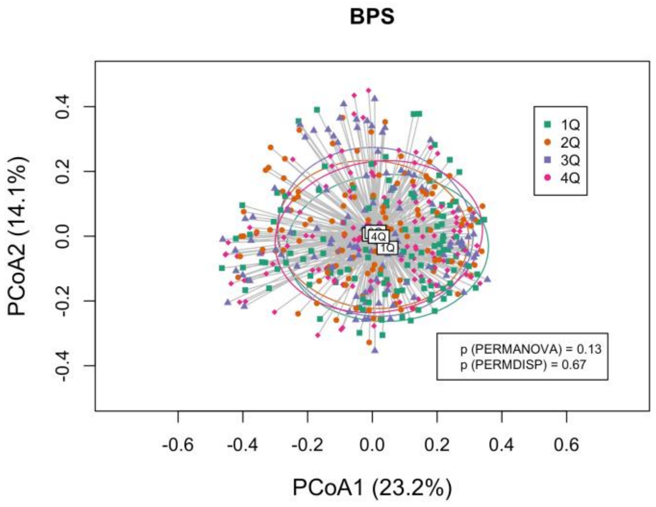

Supplementary Figure 3. Bar plot of differentially abundant genera obtained from ANCOM-BC pattern analysis. Data are represented by log fold change (shown as column). All log fold changes with p-value < 0.05 are indicated, *significant at 5% level of significance; **significant at 1% level of significance; ***significant at 0.1% level of significance. **a)** bisphenol A; **b)** bisphenol S; **c)** propylparaben; **d)** methylparaben; **e)** molar sum of parabens; **f)** ethylparaben.

a

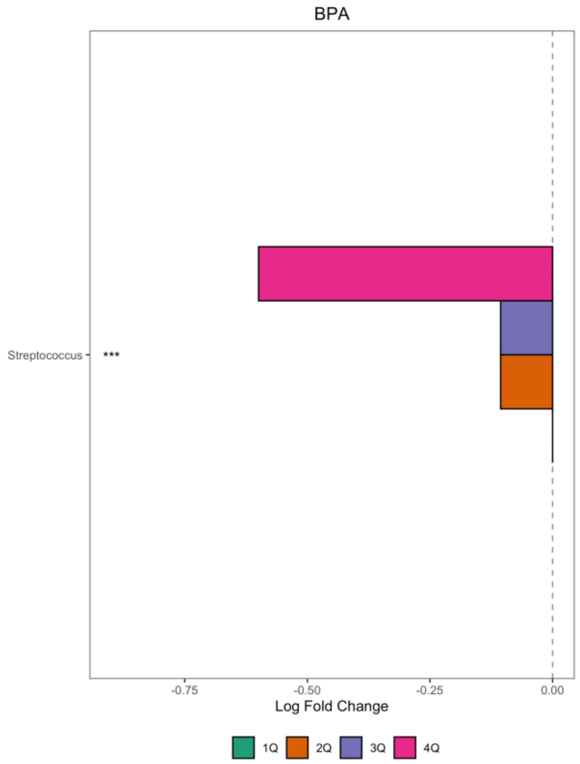

b

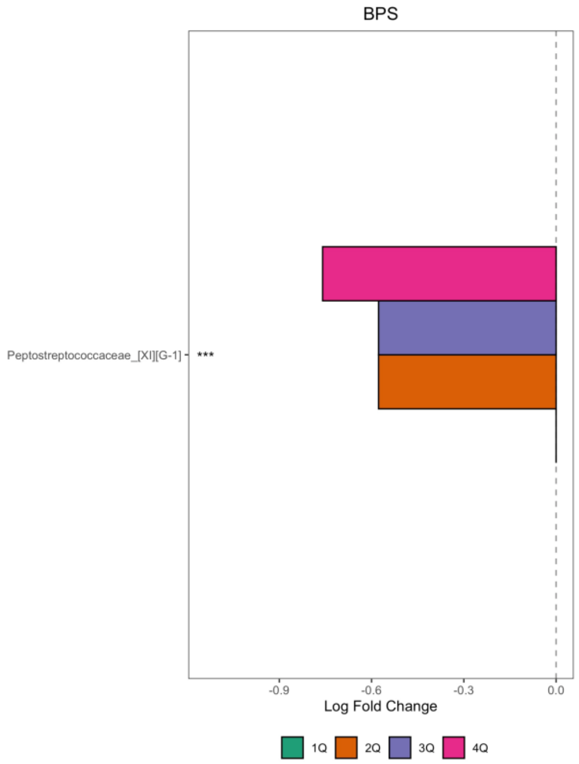

Supplementary Figure 3. Bar plot of differentially abundant genera obtained from ANCOM-BC pattern analysis. Data are represented by log fold change (shown as column). All log fold changes with p-value < 0.05 are indicated, *significant at 5% level of significance; **significant at 1% level of significance; ***significant at 0.1% level of significance. **a)** bisphenol A; **b)** bisphenol S; **c)** propylparaben; **d)** methylparaben; **e)** molar sum of parabens; **f)** ethylparaben.

c

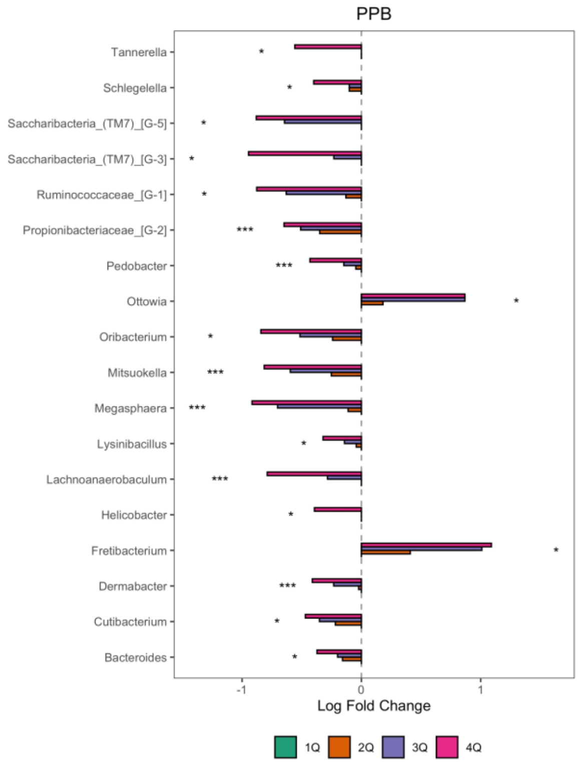

d

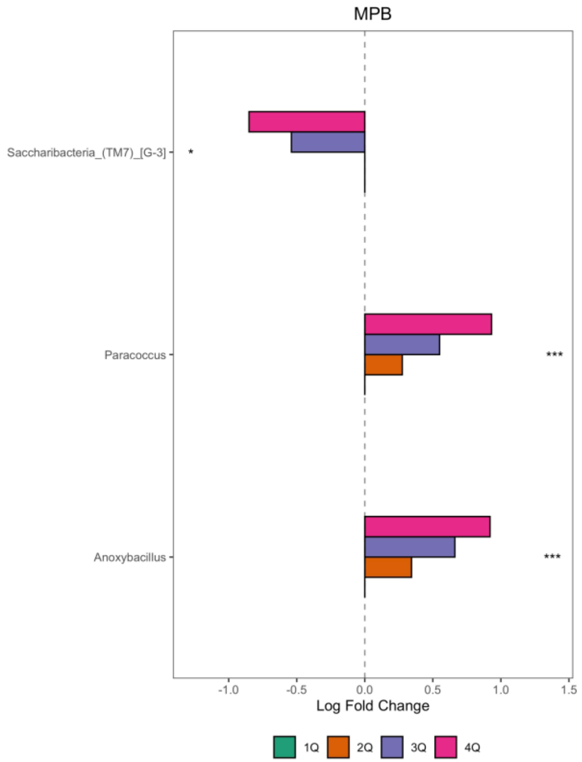

Supplementary Figure 3. Bar plot of differentially abundant genera obtained from ANCOM-BC pattern analysis. Data are represented by log fold change (shown as column). All log fold changes with p-value < 0.05 are indicated, *significant at 5% level of significance; **significant at 1% level of significance; ***significant at 0.1% level of significance. **a)** bisphenol A; **b)** bisphenol S; **c)** propylparaben; **d)** methylparaben; **e)** molar sum of parabens; **f)** ethylparaben.

e

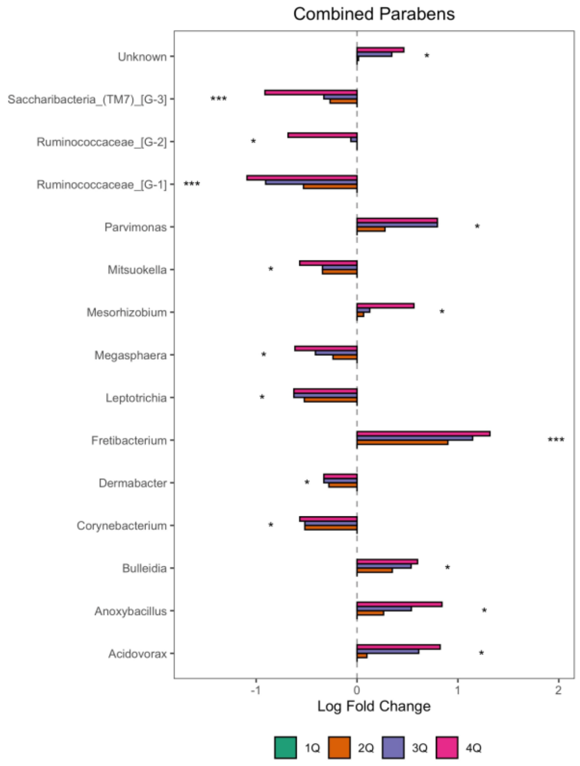

f

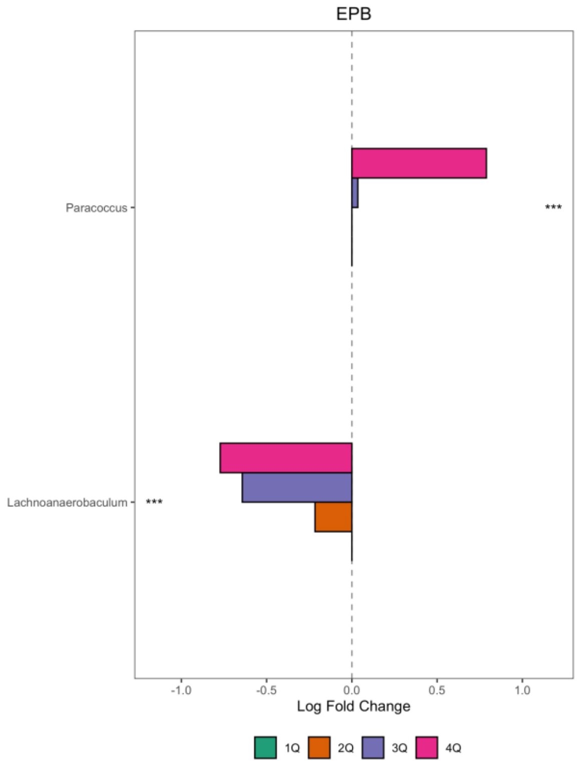

Supplement: Supplementary file 1 [file Data_Sheet_1.pdf]
